# Blocking two-component signalling enhances *Candida albicans* virulence and reveals adaptive mechanisms that counteract sustained SAPK activation

**DOI:** 10.1371/journal.ppat.1006131

**Published:** 2017-01-30

**Authors:** Alison M. Day, Deborah A. Smith, Mélanie A. C. Ikeh, Mohammed Haider, Carmen M. Herrero-de-Dios, Alistair J. P. Brown, Brian A. Morgan, Lars P. Erwig, Donna M. MacCallum, Janet Quinn

**Affiliations:** 1 Institute for Cell and Molecular Biosciences, Faculty of Medical Sciences, Newcastle University, Newcastle upon Tyne, United Kingdom; 2 Aberdeen Fungal Group, Institute of Medical Sciences, University of Aberdeen, Aberdeen, United Kingdom; Texas A&M University, UNITED STATES

## Abstract

The Ypd1 phosphorelay protein is a central constituent of fungal two-component signal transduction pathways. Inhibition of Ypd1 in *Saccharomyces cerevisiae* and *Cryptococcus neoformans* is lethal due to the sustained activation of the ‘p38-related’ Hog1 stress-activated protein kinase (SAPK). As two-component signalling proteins are not found in animals, Ypd1 is considered to be a prime antifungal target. However, a major fungal pathogen of humans, *Candida albicans*, can survive the concomitant sustained activation of Hog1 that occurs in cells lacking *YPD1*. Here we show that the sustained activation of Hog1 upon Ypd1 loss is mediated through the Ssk1 response regulator. Moreover, we present evidence that *C*. *albicans* survives SAPK activation in the short-term, following Ypd1 loss, by triggering the induction of protein tyrosine phosphatase-encoding genes which prevent the accumulation of lethal levels of phosphorylated Hog1. In addition, our studies reveal an unpredicted, reversible, mechanism that acts to substantially reduce the levels of phosphorylated Hog1 in *ypd1Δ* cells following long-term sustained SAPK activation. Indeed, over time, *ypd1Δ* cells become phenotypically indistinguishable from wild-type cells. Importantly, we also find that drug-induced down-regulation of *YPD1* expression actually enhances the virulence of *C*. *albicans* in two distinct animal infection models. Investigating the underlying causes of this increased virulence, revealed that drug-mediated repression of *YPD1* expression promotes hyphal growth both within murine kidneys, and following phagocytosis, thus increasing the efficacy by which *C*. *albicans* kills macrophages. Taken together, these findings challenge the targeting of Ypd1 proteins as a general antifungal strategy and reveal novel cellular adaptation mechanisms to sustained SAPK activation.

## Author summary

As fungi-attributed human deaths are increasing, there is an urgent need to develop new antifungal treatments. Two-component related proteins, such as the Ypd1 phosphorelay protein, have been heralded as antifungal targets as they are not found in humans and because inactivation of *YPD1* in several different fungi causes sustained SAPK activation and cell death. However, we have discovered that inactivation of *YPD1* in the major human pathogen, *Candida albicans*, actually enhances virulence. Furthermore, we reveal that this fungus adapts to the sustained activation of the Hog1 SAPK triggered by Ypd1 loss by mounting distinct mechanisms that actively reduce the level of phosphorylated Hog1. These findings question the validity of Ypd1 proteins as broad-spectrum antifungal targets and provide insights into the cellular adaptation to sustained SAPK activation.

## Introduction

*Candida albicans* is the leading cause of systemic fungal infections in humans resulting in over 400,000 deaths each year in immuno-compromised patients [1]. The ability of *C*. *albicans* to adapt to host-imposed stresses encountered during infection is an important virulence trait [2]. Central to fungal stress responses are the stress-activated protein kinases (SAPKs), which are conserved eukaryotic signalling enzymes that allow cells to adapt to environmental change [3, 4]. In *C*. *albicans*, the Hog1 SAPK is activated in response to diverse, physiologically relevant, stress conditions, and cells lacking Hog1 are acutely sensitive to such stresses [5–7]. Consistent with the vital role of the Hog1 SAPK in stress survival, *C*. *albicans* cells lacking *HOG1* display significantly attenuated virulence in systemic, commensal, and phagocyte infection models [8–11].

All SAPK activation mechanisms reported to date result in the phosphorylation of conserved threonine and tyrosine residues located within the TGY motif of the catalytic domain of the kinase [3]. Such pathways are tightly regulated as the nature of the response is dependent on the extent and period of SAPK activation. For example, in the model yeast *Saccharomyces cerevisiae*, transient activation of the Hog1 SAPK is vital to survive osmotic stress [4], whereas sustained activation triggers programmed cell death [12]. Similarly in human cells transient activation of the p38 SAPK promotes stress-induced gene expression and cellular proliferation [13], whereas sustained SAPK activation triggers apoptosis [14]. In contrast, much less is known regarding the regulation and cellular consequences of sustained SAPK activation in *C*. *albicans*.

**Fig 1:**
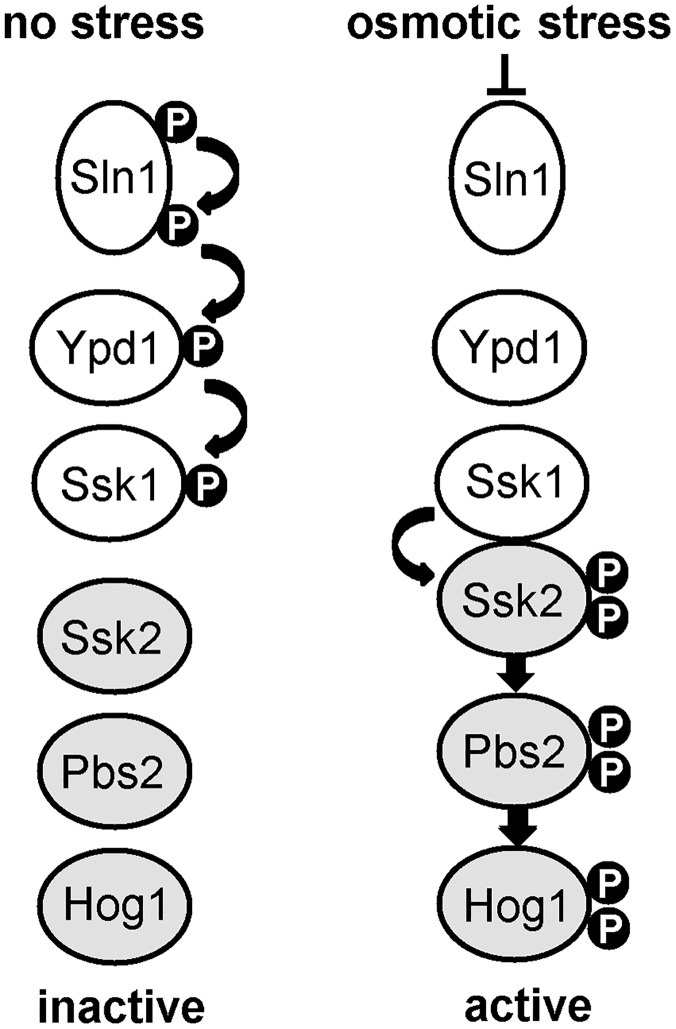
Overview of two component-mediated regulation of the Hog1 SAPK in *S*. *cerevisiae*. Under non-stress conditions the Sln1 histidine kinase autophosphorylates, and this phosphate is transferred via the Ypd1 phosphorelay protein to the Ssk1 response regulator protein. Following osmotic stress Sln1 is inactivated, thus halting the phosphorelay which culminates in unphosphorylated Ssk1 which is a potent activator of the Ssk2 MAPKKK. This results in the phosphorylation and activation of the downstream Hog1 SAPK.

Despite the availability of several antifungal drugs, the high mortality rate associated with *C*. *albicans* systemic infections and the emergence of drug resistant strains highlights the urgent clinical need for new anti-fungal therapies [15]. Although Hog1 is an essential virulence determinant in *C*. *albicans*, the conservation with highly related SAPKs in human cells suggests that Hog1 itself may be unsuitable as an antifungal target. Instead, there has been much interest in identifying fungal-specific regulators of SAPKs as potential drug targets. Candidate targets include two-component related phosphorelay systems which constitute an important mechanism employed by fungi, but not mammals, to sense and relay specific stress signals to SAPK modules [16]. In *S*. *cerevisiae*, this system is comprised of a hybrid histidine kinase (Sln1), an intermediary phosphorelay protein (Ypd1), and a response regulator protein (Ssk1) (Fig 1). Following osmotic stress, the Sln1 histidine kinase is inactivated, which halts phosphorelay through Ypd1, and consequently leads to the rapid dephosphorylation of Ssk1 [17]. Dephosphorylated Ssk1 is a potent activator of the Ssk2/Ssk22 MAPKKKs which regulate Hog1 activation [18, 19]. Significantly, loss of either Sln1 or Ypd1 function in *S*. *cerevisiae* is lethal [20], due to the accumulation of unphosphorylated Ssk1 and the resulting sustained Hog1 activation which triggers apoptosis-mediated cell death [12]. It is likely that sustained Hog1 activation can also not be tolerated in the human fungal pathogen, *Cryptococcus neoformans*, as Ypd1 is essential for the viability of cells containing Hog1 [21]. The essential nature of Ypd1 in these fungi, and the many reports illustrating the importance of two-component proteins in fungal pathogenicity, has fuelled interest in targeting Ypd1 for antifungal drug development (reviewed in [22]).

*C*. *albicans* has seven two-component proteins; three histidine kinases (Sln1, Chk1, Nik1), three response regulators (Ssk1, Skn7, Crr1/Srr1), and a single phosphorelay protein (Ypd1) [23]. In *S*. *cerevisiae*, Ypd1 plays a pivotal role in mediating all phosphorelay events from the upstream histidine kinases to the downstream Ssk1 and Skn7 response regulators [17, 24], which supports the concept that Ypd1 is an appropriate antifungal target. Hence, in this study, we investigated the impact of inactivating Ypd1 upon stress signalling and virulence of *C*. *albicans*. As reported recently, we found that *C*. *albicans* can survive deletion of *YPD1* [25]. Here we extend this finding by illustrating that *C*. *albicans* survives the sustained SAPK activation following Ypd1 loss by evoking multiple mechanisms to reduce the level of phosphorylated Hog1. Furthermore, we demonstrate that inactivation of Ypd1 during infection actually increases the virulence of *C*. *albicans* in a number of infection models, revealing that Ypd1 may not be a suitable target for anti-fungal drug development.

## Results

### Repression or deletion of *YPD1* in *C*. *albicans* induces sustained Hog1 activation

**Fig 2:**
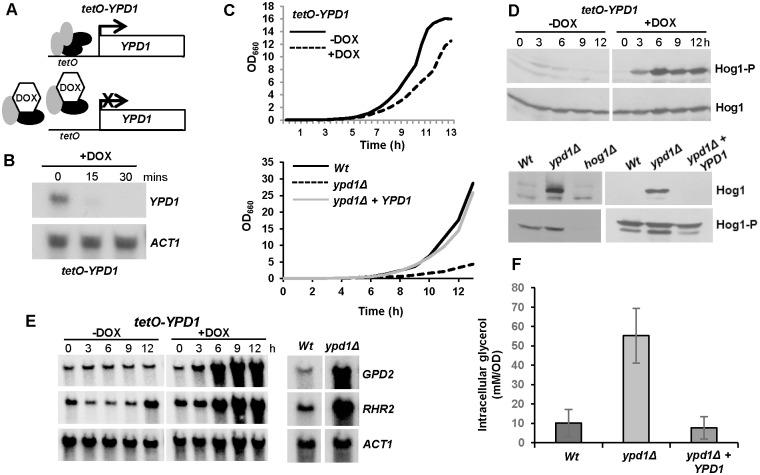
*C*. *albicans* cells lacking *YPD1* exhibit hyperactivation of Hog1 but are viable. (A) Strategy to control *YPD1* expression. One *YPD1* allele was deleted, and the remaining allele placed under the control of the *E*. *coli tet* operator (*tetO*) in strain THE1 to generate strain *tetO-YPD1* (JC1586). THE1 cells express an *E*. *coli tet* repressor–*S*. *cerevisiae* Hap4 activation domain fusion protein. In the absence of doxycycline (DOX), this fusion protein binds as a dimer to the *tet* operator resulting in transcriptional activation. However, doxycycline prevents dimerisation of the fusion protein and blocks transcription. (B) Doxycycline treatment inhibits *YPD1* expression. Northern analysis of *YPD1* and *ACT1* (control) transcript levels in *tetO-YPD1* cells treated with doxycycline for the indicated times. (C) Repression or deletion of *YPD1* results in a slow growth phenotype. Growth analysis of *tetO-YPD1* cells, untreated or treated with doxycycline (top panel), and wild-type (Wt, JC21), *ypd1Δ* (JC2001) and *ypd1Δ+YPD1* (JC2002) cells (bottom panel). (D) Repression or deletion of *YPD1* results in constitutive phosphorylation of Hog1. Western blot analysis of whole cell extracts isolated from *tetO-YPD1* cells following treatment with doxycycline for the indicated times, or from exponentially growing wild-type (Wt), *ypd1Δ* and *ypd1Δ+YPD1* cells. Blots were probed for phosphorylated Hog1 (Hog1-P), stripped and reprobed for total Hog1 (Hog1). (E) Repression or deletion of *YPD1* results in high levels of *GPD2* and *RHR2* expression. RNA was isolated from *tetO-YPD1* cells, treated with or without doxycycline for the indicated times, or wild-type and *ypd1Δ* cells, and analyzed using gene-specific probes with *ACT1* as a loading control. (F) Deletion of *YPD1* results in increased intracellular glycerol levels. The mean ± SD is shown for 3 biological replicates.

*C*. *albicans* contains a single homologue of the *S*. *cerevisiae* phosphorelay protein Ypd1 [26]. Although deletion of Ypd1 results in a lethal phenotype in both *S*. *cerevisiae* and *C*. *neoformans* [20, 21], a recent study revealed that *YPD1* is not an essential gene in *C*. *albicans*, with *ypd1Δ* cells instead displaying a slow growth phenotype [25]. To investigate this further we created a *C*. *albicans* strain, *tetO-YPD1* (Fig 2A), in which one allele of *YPD1* was deleted and the remaining allele placed under the control of a doxycycline-repressible promoter [27]. Northern analysis confirmed that treatment of *tetO-YPD1* cells with doxycycline caused a rapid decrease in *YPD1* mRNA levels (Fig 2B). However, whilst repression of *YPD1* expression did result in a slower growth rate (Fig 2C, upper panel), the cells were viable. Furthermore, consistent with the previous study [25], we were able to generate a viable homozygous *ypd1Δ* null mutant which displayed a slower growth rate compared to wild-type cells (Fig 2C, lower panel).

Deletion of *YPD1* is lethal in *S*. *cerevisiae* due to constitutive SAPK activation. Consistent with previous findings [25], we found that repression of *YPD1* expression in *tetO-YPD1* cells (Fig 2D, upper panel; S1 Fig), or deletion of *YPD1* (Fig 2D, lower panel), also stimulated high levels of Hog1 phosphorylation in *C*. *albicans*. Together these data indicate that the inhibitory effect of *YPD1* on SAPK activation in *S*. *cerevisiae* is conserved in *C*. *albicans*. However, it was possible that the phosphorylated Hog1 detected did not result in activation of Hog1-dependent downstream events. To test this possibility the expression levels of the Hog1-dependent genes, *GPD2* and *RHR2* [28], important for glycerol biosynthesis were examined upon repression or deletion of *YPD1* (Fig 2E). Both genes were found to be up-regulated and, furthermore, as expected, increased intracellular glycerol concentrations were observed in *ypd1Δ* cells in the absence of stress (Fig 2F). Thus collectively, these data confirm that the phosphorylated Hog1 kinase triggered by inactivation of Ypd1 in *C*. *albicans* is active. However, in contrast to *S*. *cerevisiae* and *C*. *neoformans* constitutive Hog1 activation does not result in loss of viability in *C*. *albicans*.

### Phenotypes associated with loss of Ypd1 are dependent on Hog1 and Ssk1

**Fig 3:**
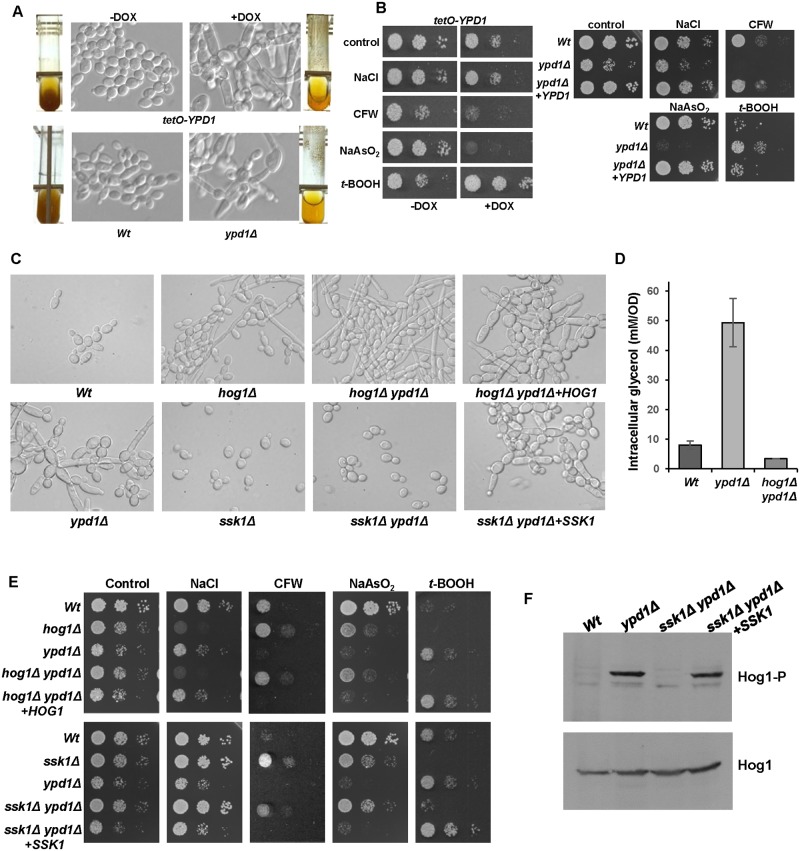
Phenotypes associated with loss of Ypd1 are dependent on Hog1 and Ssk1. (A) Repression or deletion of *YPD1* triggers flocculation and a swollen pseudohyphal filamentous phenotype. Micrographs of *Wt*, *ypd1Δ*, and *tetO-YPD1* cells plus or minus doxycycline (DOX) grown overnight in rich media. Images of culture tubes demonstrate the rapid sedimentation rate of cells lacking *YPD1*. (B) Repression or deletion of *YPD1* results in pleiotropic stress phenotypes. 10^4^ cells, and 10-fold dilutions thereof, of exponentially growing *tetO-YPD1* cells, or wild-type (*Wt*), *ypd1Δ* and *ypd1Δ+YPD1* cells, were spotted onto rich media plates (plus or minus DOX for *tetO-YPD1* cells) containing NaCl (1.0 M), calcofluor white (CFW, 30 μg/ml), NaAsO_2_ (1.5 mM) and *t*-BOOH (2 mM), and incubated at 30°C for 24h. (C) The morphological defects exhibited by *ypd1Δ* cells are dependent on Hog1 and Ssk1. Micrographs of wild-type (*Wt*), *ypd1Δ*, *hog1Δ* (JC50), *ssk1Δ* (JC1552), *hog1Δ ypd1Δ* (JC1475) *hog1Δypd1Δ*+*HOG1* (JC1478), *ssk1Δ ypd1Δ* (JC1683), and *ssk1Δ ypd1Δ*+*SSK1* (JC1704) cells. (D) The high glycerol levels in *ypd1Δ* cells are dependent on Hog1. The mean ± SD is shown for 3 biological replicates. (E) The stress phenotypes exhibited by *ypd1Δ* cells are dependent on Hog1 and Ssk1. Exponentially growing strains were spotted onto rich media plates containing the additives detailed in B above, and incubated at 30°C for 24h. (F) The sustained Hog1 activation in *ypd1Δ* cells is dependent on Ssk1. Western blots depicting basal levels of Hog1 phosphorylation in the indicated strains. Blots were probed for phosphorylated Hog1 (Hog1-P), stripped and reprobed for total Hog1 (Hog1).

Analyses of cells with loss of Ypd1 function, either by repression or deletion of the *YPD1* gene, revealed identical morphological abnormalities and stress-resistance profiles. For example, loss of *YPD1* resulted in swollen pseudohyphal-like cells (Fig 3A), possibly due to the increased intracellular levels of the osmolyte glycerol that occurs upon inactivation of Ypd1 (Fig 2F). In addition, as reported previously [25], cells lacking Ypd1 were highly flocculent as demonstrated by their rapid sedimentation rate (Fig 3A). Interestingly, cells lacking Ypd1 displayed acute sensitivity to sodium arsenite, increased resistance to the organic peroxide *tert*-butyl hydroperoxide (*t*-BOOH), and wild-type levels of resistance to osmotic stress (Fig 3B). Loss of *YPD1* also resulted in increased sensitivity to the cell wall perturbing agent calcofluor white (Fig 3B). This is consistent with Hog1 activation in *ypd1Δ* cells (Fig 2D), as *hog1Δ* cells display significant resistance to this drug [6].

Loss of Ypd1 function is predicted to perturb phosphorelay to all three response regulator proteins in *C*. *albicans*; Ssk1, Skn7 and Crr1/Srr1 [23, 29, 30]. In *S*. *cerevisiae* it is the accumulation of the unphosphorylated Ssk1 response regulator which triggers hyperactivation of the Hog1 SAPK [18]. Hence, to investigate which phenotypes associated with Ypd1 loss in *C*. *albicans* were due to Ssk1-mediated activation of Hog1, *hog1Δ ypd1Δ* and *ssk1Δ ypd1Δ* double mutant strains were created. All of the *ypd1Δ*-associated phenotypes, described above, were found to be dependent on both Hog1 and Ssk1. For example, the swollen pseudohyphal-like morphology associated with *ypd1Δ* mutant cells was repressed in the absence of either *HOG1* or *SSK1* (Fig 3C). In fact, the *hog1Δ ypd1Δ* double mutant cells instead displayed the morphological defects characteristic of *hog1Δ* cells (Fig 3C). The high glycerol levels characteristic of *ypd1Δ* cells were dependent on Hog1 (Fig 3D), and the stress-phenotypes associated with deletion of *YPD1* were not maintained in *hog1Δ ypd1Δ* or *ssk1Δ ypd1Δ* double mutant cells (Fig 3E). Indeed, in all the conditions examined, cells lacking *HOG1* and *YPD1* displayed similar stress phenotypes as *hog1Δ* cells, and *ssk1Δ ypd1Δ* cells were phenotypically similar to *ssk1Δ* cells. Importantly, confirming the link between Ypd1 and Hog1 activity, reintegration of *HOG1* or *SSK1* into the *hog1Δ ypd1Δ* and *ssk1Δ ypd1Δ* mutants, respectively, resulted in cells that were phenotypically identical to the *ypd1Δ* strain (Fig 3C and 3E). Furthermore, the hyper-phosphorylation of Hog1 detected in *ypd1Δ* cells was absent in *ssk1Δ ypd1Δ* cells, but was restored upon reintegration of *SSK1* (Fig 3F). This result confirms that Ssk1 is essential for the high basal level of Hog1 phosphorylation in *ypd1Δ* cells. Taken together, these results are consistent with the model that accumulation of unphosphorylated Ssk1 triggers the *ypd1Δ*-dependent sustained activation of Hog1 in *C*. *albicans* and, moreover, that this activation underlies the morphological and stress phenotypes associated with loss of Ypd1.

### *C*. *albicans* survives sustained activation of Hog1 by induction of the negative regulators Ptp2 and Ptp3

**Fig 4:**
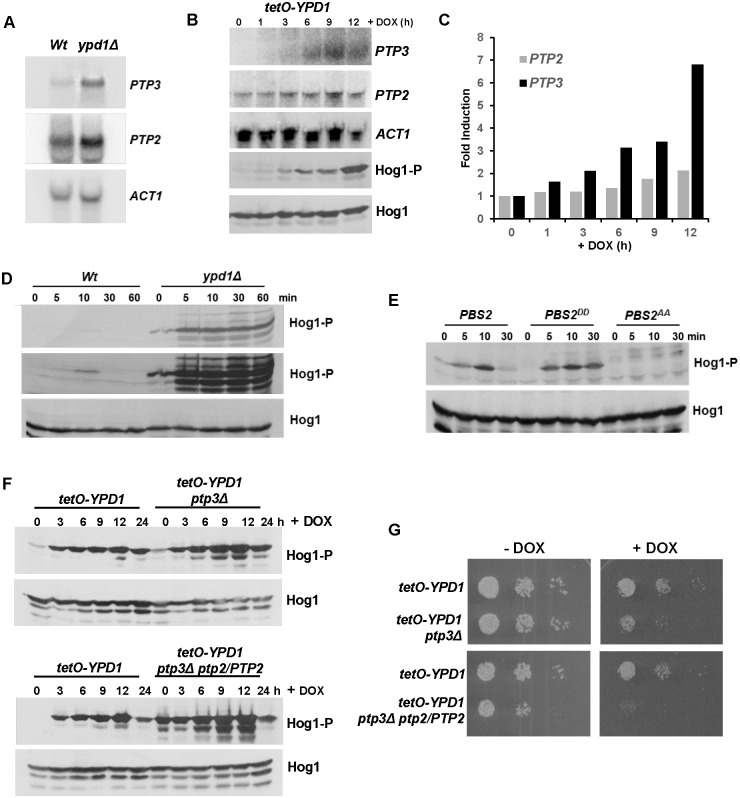
*C*. *albicans* cells adapt to loss of Ypd1 function by inducing negative regulators of Hog1. (A) *PTP2* and *PTP3* are induced in *ypd1Δ* cells. Northern blot analysis of *PTP2* and *PTP3* expression in exponentially growing *Wt* (JC21 and *ypd1Δ* (JC2001) cells. *ACT1* was used as a loading control. (B) The kinetics of *PTP3* and *PTP2* are similar to that of Hog1 activation following doxycycline treatment of *tetO-YPD1* cells. Northern blot analyses of *PTP2* and *PTP3* expression, and western blot analysis of Hog1 phosphorylation in *tetO-YPD1* cells (JC1586) following treatment with doxycycline for the indicated times. (C) Quantification of *PTP3* and *PTP2* induction following doxycycline treatment of *tetO-YPD1* cells. (D) The tyrosine phosphatase inhibitor, arsenite, further activates Hog1 in *ypd1Δ* cells. Western blot analysis of whole cell extracts isolated from exponentially growing *Wt* and *ypd1Δ* cells treated with 5mM NaAsO_2_ for the specified times. Duplicate blots were probed for phosphorylated Hog1 (Hog1-P) or total Hog1 (Hog1) levels. A darker exposure of the Hog1-P blot is included (middle panel) to show the level of Hog1-P observed in *Wt* cells following arsenite treatment. (E) Hog1 is activated by arsenite in *PBS2*^*DD*^ cells. Western blot analysis of whole cell extracts isolated from exponentially growing *PBS2* (JC112), *PBS2*^*AA*^ (JC126) and *PBS2*^*DD*^ (JC124) cells after treatment with 5mM NaAsO_2_ for the specified times. Blots were probed for phosphorylated Hog1 (Hog1-P), stripped, and reprobed for total Hog1 (Hog1) levels. (F) Deletion of *PTP* genes trigger greater activation of Hog1 following repression of *YPD1*. Western blot analysis of whole cell extracts isolated from *tetO-YPD1*, *tetO-YPD1 ptp3Δ* (JC2188) and *tetO-YPD1 ptp3Δ PTP2/ptp2* (JC2195) cells following treatment with doxycycline for the indicated times. Duplicate blots were probed for phosphorylated Hog1 (Hog1-P) or total Hog1 (Hog1) levels. (G) Deletion of *PTP* genes impairs cell growth following repression of *YPD1*. 10^4^ cells, and 10-fold dilutions thereof, of the indicated strains were spotted onto rich media plates plus or minus DOX, and incubated at 30°C for 24h.

We next investigated the molecular mechanism underlying the ability of *C*. *albicans* to survive sustained Hog1 activation. In *S*. *cerevisiae*, the lethality associated with *YPD1* loss can be by-passed by the artificial over-expression of either of the protein tyrosine phosphatases, Ptp2 or Ptp3, which normally dephosphorylate and negatively regulate Hog1 activity [31, 32]. Indeed, *YPD1* (tyrosine phosphatase dependent) was initially identified in a synthetic lethal screen for *S*. *cerevisiae* mutants whose growth was dependent on the expression of *PTP2* [33]. Similarly, Ptp2 in *C*. *neoformans* [34] and Ptp2 and Ptp3 in *C*. *albicans* [35], have been reported to negatively regulate the respective Hog1 SAPK pathways in these pathogenic fungi. Hence, it was possible that the ability of *C*. *albicans ypd1Δ* cells to retain viability was linked to the activity of the Ptp2 and Ptp3 phosphatases. Strikingly, we found that the expression of *PTP3* is significantly induced in *ypd1Δ* cells compared to wild-type cells, and *PTP2* is also up-regulated albeit to a lesser extent (Fig 4A). Similar findings were also observed upon doxycycline-mediated repression of *YPD1* in *tetO-YPD1 cells* (Fig 4B and 4C). Significant induction of *PTP3* occurred with similar kinetics as the increase in Hog1 activation, and some induction of *PTP2* was also evident (Fig 4B and 4C). Previous studies revealed that arsenite is a potent inhibitor of protein tyrosine phosphatases that regulate SAPK pathways in both yeast and humans [36–38]. Interestingly, *C*. *albicans* cells lacking Ypd1 are acutely sensitive to arsenite (Fig 3B), raising the possibility that arsenite-mediated inhibition of Ptp2 and/or Ptp3 causes catastrophic levels of Hog1 phosphorylation and ultimately cell death. Indeed, in agreement with this hypothesis, sodium arsenite treatment massively increased Hog1 phosphorylation in *ypd1Δ* but not wild-type cells (Fig 4D). To confirm that arsenite primarily activates Hog1 through phosphatase inhibition, we next examined Hog1 phosphorylation in cells expressing a mutant version of the MAPKK Pbs2 (Pbs2^DD^), where the activating phosphorylation sites of Pbs2 are mutated to phosphomimetic aspartate residues. The Pbs2^DD^ mutant protein yields basal activation of Hog1 but prevents further upstream signalling events to the SAPK [10]. Consistent with arsenite-dependent inhibition of Hog1-specific phosphatase(s), significant induction of Hog1 phosphorylation was observed in Pbs2^DD^ cells following arsenite treatment (Fig 4E). Furthermore, this arsenite-induction of Hog1 requires Pbs2 activity as arsenite-induced Hog1 phosphorylation does not occur in cells expressing an inactive Pbs2^AA^ kinase where the activating phosphorylation sites of Pbs2 are mutated to alanine residues mimicking hypophosphorylation (Fig 4E, compare *PBS2* and *PBS2*^*DD*^ with the *PBS2*^*AA*^ lanes). Thus, these results strongly suggest that the ability of *C*. *albicans* cells to survive sustained activation of Hog1 upon loss of Ypd1 function is due to the action of Ptp phosphatases that reduce the levels of phosphorylated Hog1.

To test this directly, we sought to delete both *PTP2* and *PTP3* in *tetO-YPD1* cells with the prediction that doxycycline-mediated repression of *YPD1* in this background would result in catastrophic levels of Hog1 activation and cell death. Initially we focused on deleting *PTP3* as this gene shows the greatest induction upon loss of *YPD1* (Fig 4A and 4B). Deletion of *PTP3* in *tetO-YPD1* cells resulted in notably higher levels of Hog1 phosphorylation upon repression of *YPD1* expression (Fig 4F). Moreover, deletion of *PTP3* had a dramatic impact on the growth of *tetO-YPD1* cells upon doxycycline-mediated repression of *YPD1*, but not in the absence of doxycycline (Fig 4G). These results support the model that induction of *PTP3* promotes *C*. *albicans* survival following Ypd1 loss by limiting Hog1 phosphorylation. However, repression of *YPD1* in *ptp3Δ* cells did not result in a lethal phenotype which is consistent with previous findings that Ptp2 and Ptp3 function redundantly to regulate *C*. *albicans* Hog1 [35]. Upon attempting to generate *tetO-YPD1 ptp3Δ ptp2Δ* cells we found that only one copy of *PTP2* could be deleted. This was unexpected as a *ptp2Δ ptp3Δ* double mutant has previously been characterised [35]. Strikingly, however, *tetO-YPD1 ptp3Δ ptp2/PTP2* cells exhibited a much higher basal level of Hog1 activation than that seen cells lacking *PTP3* (Fig 4F). Following doxycycline-mediated repression of *YPD1* in *ptp3Δ ptp2/PTP2* cells, further substantial increases in Hog1 phosphorylation were detected compared to that observed in *tetO-YPD1 ptp3Δ* cells (Fig 4F). Indeed, consistent with the additive effect of deleting one copy of *PTP2*, the growth of *tetO-YPD1 ptp3Δ ptp2/PTP2* cells was barely detectable upon repression of *YPD1* expression (Fig 4G). However, it is important to note that such cells also display a slow growth phenotype under −DOX conditions when *YPD1* is expressed (Fig 4G). Nonetheless, taken together, these data support the model that the induction of *PTP3* and *PTP2*, together with the basal expression of *PTP2*, facilitate *C*. *albicans* survival following loss of the Ypd1 phosphorelay protein.

### *C*. *albicans* cells lacking Ypd1 adapt to become phenotypically similar to wild-type cells

**Fig 5:**
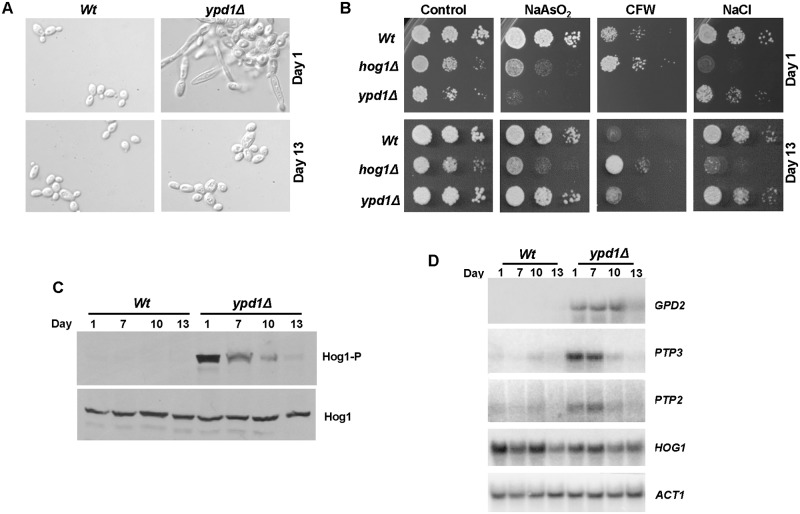
*C*. *albicans* adapts to long term Ypd1 loss by lowering Hog1 activity. (A) *ypd1Δ* cells become morphologically similar to wild-type cells over time. Micrographs of exponentially growing *Wt* (JC21) and *ypd1Δ* (JC2001) cells taken from rich media plates after 1 or 13 days. (B) *ypd1Δ* cells gradually accumulate stress phenotypes characteristic of wild-type cells. Approximately 10^4^ cells, and 10-fold dilutions thereof, of exponentially growing *Wt*, *hog1Δ* (JC50) and *ypd1Δ* cells taken from rich media plates after 1 or 13 days were spotted onto plates containing; NaAsO_2_ (1.5 mM), calcofluor white (CFW 30 μg/ml) and NaCl (0.5 M). Plates were incubated at 30°C for 24 hrs. (C) Hog1 phosphorylation is not sustained in *ypd1Δ* cells over time. Western blot analysis of whole cell extracts isolated from exponentially growing *Wt* and *ypd1Δ* cells taken from rich media plates after the number of days indicated. Blots were probed for phosphorylated Hog1 (Hog1-P), stripped and reprobed for total Hog1 (Hog1). (D) *PTP2*, *PTP3*, and *GPD2* expression is not sustained in *ypd1Δ* cells. Northern blot analysis of the indicated genes in exponentially growing *Wt* and *ypd1Δ* cells taken from plates after the number of days indicated. *ACT1* was used as a loading control.

Although *C*. *albicans* can clearly tolerate sustained Hog1 activation, cells lacking *YPD1* display reduced fitness compared to wild-type cells (Fig 2C). Strikingly, however, we noted that *ypd1Δ* cells, when maintained on rich media plates, lost the morphological abnormalities associated with inactivation of Ypd1 function. Specifically, the swollen pseudohyphal morphology observed in freshly isolated *ypd1Δ* cells (Day 1), was largely replaced with normal budding cells after incubation on rich media plates for 13 days (Fig 5A). Because of these findings we examined whether the stress phenotypes associated with *YPD1* loss also changed over time. In agreement with our previous findings (Fig 3), freshly isolated *ypd1Δ* cells (Day 1) exhibited a slow growth rate (indicated by small colony size), and increased sensitivity to sodium arsenite and calcofluor white. However, by day 13 the slow growth phenotype was lost, and the sensitivity to sodium arsenite and calcofluor white mimicked that exhibited by wild-type cells (Fig 5B). Consistent with this loss of morphological and stress sensitive phenotypes, we found that the high basal level of Hog1 phosphorylation, triggered by loss of *YPD1*, decreased over a 13 day period (Fig 5C). Hog1 phosphorylation levels were significantly reduced by day 10, and by day 13 levels were similar to that seen in wild-type cells. Consistent with a reduction in Hog1 activation, the high basal level of expression of the Hog1 target gene, *GPD2*, also declined over time (Fig 5D). The decrease in Hog1 phosphorylation was not due to a reduction in Hog1 protein and/or *HOG1* mRNA levels which remained constant over the 13 day experiment (Fig 5C and 5D). This suggests that the sustained SAPK phosphorylation caused by loss of Ypd1 triggers adaptation within the cell that results in lower levels of activated Hog1.

As we had found the negative regulators *PTP3* and *PTP2* to be induced in *ypd1Δ* cells, we asked whether a further induction in their expression could contribute to the adaptation mechanism resulting in the time-dependent decline in Hog1 phosphorylation levels. In agreement with our previous findings (Fig 4A), we found *PTP3* and *PTP2* to be up-regulated in *ypd1Δ* cells at day 1 and this was maintained at day 7 (Fig 5B). However, by day 10 the levels of *PTP3* and *PTP2* in *ypd1Δ* cells had returned to wild-type levels. Hence, although *C*. *albicans* appears to adapt to sustained SAPK activation in the short term by triggering the up-regulation of *PTP2* and *PTP3*, this up-regulation is temporary. Indeed, the induction of *PTP2* and *PTP3* is only observed in cells in which significant levels of Hog1 activation are seen (compare Fig 5C and 5D). This suggests that *C*. *albicans* adapts to Hog1 activation in the long term by via another mechanism(s) independent of Ptp2 and Ptp3. Although the specific adaptation mechanism has not been identified, these results illustrate that *C*. *albicans* cells adapt to *YPD1* loss over time by reducing the levels of phosphorylated Hog1 such that the negative effects of sustained SAPK activation are ablated, and *ypd1Δ* cells become phenotypically similar to wild-type cells.

### The reduction in Hog1 phosphorylation levels triggered by sustained SAPK activation can be reversed following stress exposure

**Fig 6:**
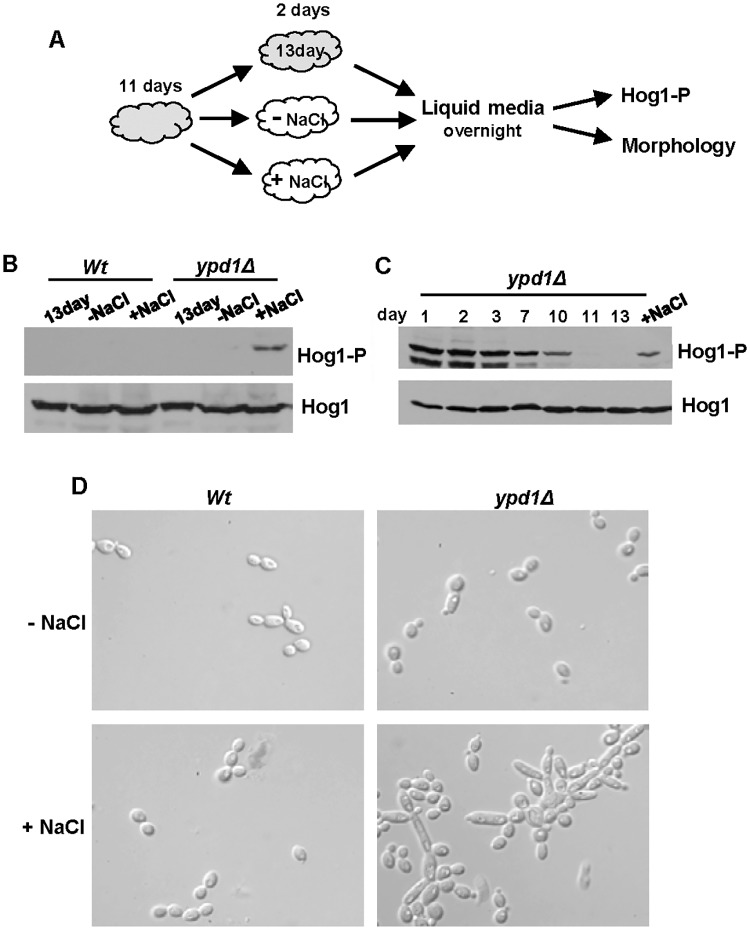
The reduction of Hog1 phosphorylation following loss of Ypd1 function can be reversed by stress exposure. (A) Experimental overview. Freshly isolated cells were incubated on rich solid media for 11 days (depicted in grey) and then either re-streaked onto fresh rich media with (+NaCl) or without (-NaCl) 0.3M NaCl (depicted in white) and incubated for a further 2 days, or maintained on the original plate for a further 2 days (13 day). Cells were then cultured overnight in liquid rich media lacking NaCl, and Hog1 phosphorylation and cellular morphology examined. (B) Western blot analysis of both phosphorylated and total Hog1 levels in *Wt* (JC21) and *ypd1Δ* (JC2001) cells treated as described in A. (C) Western blot analysis of whole cell extracts isolated from exponentially-growing *ypd1Δ* cells taken either from rich media plates after the number of days indicated, or after being re-streaked on rich media with NaCl (+NaCl) as described in A. Duplicate blots were probed for phosphorylated Hog1 (Hog1-P) or total Hog1 (Hog1) levels. (D) Micrographs of exponentially-growing *Wt* and *ypd1Δ* strains treated as described in A.

We asked whether the reduction in Hog1 phosphorylation levels following long-term sustained Hog1 activation was irreversible. Cells lacking *ypd1Δ* that had been maintained for 11 days on solid rich media, were then either kept on this media (13 day) or patched onto fresh media in the absence (-NaCl) or presence (+NaCl) of 0.3M NaCl, a stress condition known to transiently activate Hog1 (Fig 6A). Cells were taken from these plates after 2 days, sub-cultured in liquid media, and phosphorylation and total levels of Hog1 examined (Fig 6A). As expected, a high basal level of Hog1 phosphorylation was absent in *ypd1Δ* cells at 13 days (Fig 6B). However, passage of *ypd1Δ* cells over plates containing NaCl, but not lacking NaCl, resulted in a restoration of Hog1 phosphorylation (Fig 6B). To determine whether NaCl treatment could fully restore Hog1 activation in *ypd1Δ* cells, we compared the level of Hog1 phosphorylation in *ypd1Δ* cells over the 13 day time course with that seen following passage over NaCl plates. Whilst exposure of *ypd1Δ* cells to NaCl does restore a high basal level of Hog1 phosphorylation, this is not to the same level as that seen in day 1 samples (Fig 6C). Therefore, these data reveal that a transient exposure of *ypd1Δ* cells to osmotic stress can partially over-ride the adaptation mechanism(s) that prevents sustained Hog1 phosphorylation. Wild-type cells did not exhibit an increase in Hog1 phosphorylation after passage over media containing NaCl, illustrating that the phosphorylation observed in *ypd1Δ* cells is due to a re-establishment of sustained Hog1 activation, rather than stress-induced activation of the SAPK (Fig 6B). Furthermore, the restoration of sustained Hog1 phosphorylation in *ypd1Δ* cells, resulted in the return of a swollen pseudohyphal morphology (Fig 6D). Collectively, the results indicate that the cellular adaptation mechanisms that reduce Hog1 activation in cells lacking *YPD1* can be partially over-ridden in the presence of stress that requires Hog1 function.

### Repression of *YPD1* expression during infection potentiates *C*. *albicans* virulence

The lethality associated with the deletion of *YPD1* in *S*. *cerevisiae* and the fungal pathogen *C*. *neoformans* [20, 21], has led to much interest in this phosphorelay protein family as a potential prime antifungal target [22]. Hence, we next examined the impact of doxycycline-mediated repression of *YPD1* on *C*. *albicans* virulence. The doxycycline-regulatable gene expression system has been successfully used to control *C*. *albicans* gene expression during infection in both a mouse model of systemic candidiasis [27, 39], and in a *Caenorhabditis elegans* infection model [40]. Furthermore, repression of *YPD1* expression after infection has three clear advantages over testing the virulence of *ypd1Δ* cells directly. Firstly, this avoids the problems of accurately obtaining an inoculum size with the highly flocculent and filamentous *ypd1Δ* strain. Secondly, complications arising from lack of efficient dissemination of a highly flocculent strain are circumvented. Finally, doxycycline-mediated repression of *YPD1* following infection more closely mimics the drug-induced inactivation of a particular fungal target following infection.

**Fig 7:**
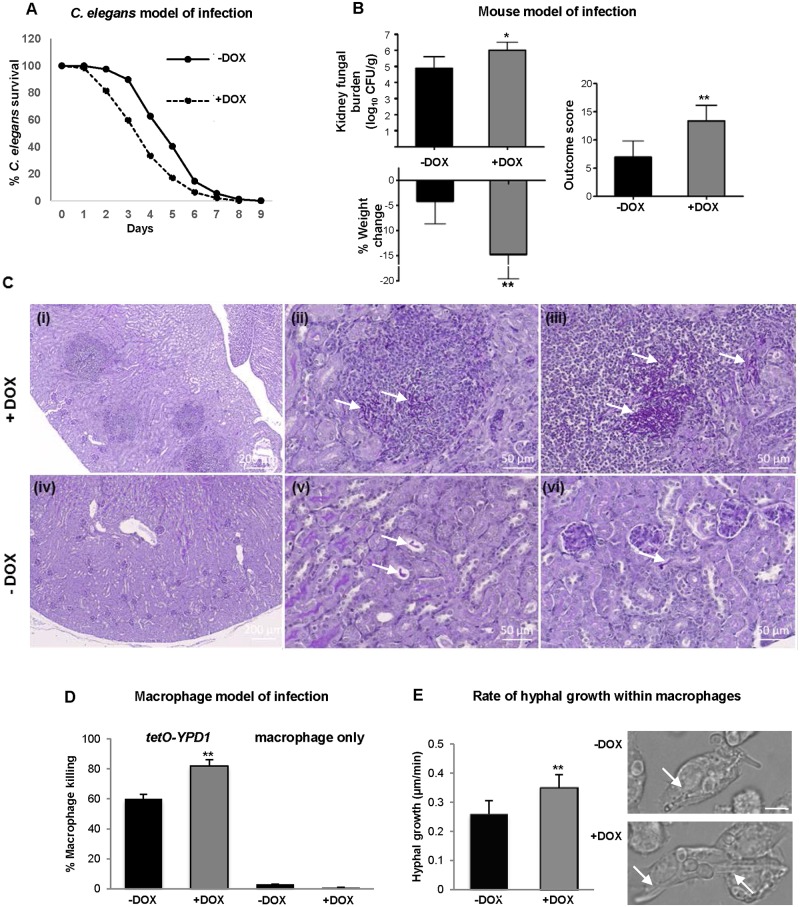
Repression of *YPD1* expression during infection potentiates *C*. *albicans* virulence. (A) *C*. *elegans model of infection*. Nematodes were infected with the conditional *tetO-YPD1* strain (JC1586) and transferred to liquid medium either with (+DOX) or without (-DOX) doxycycline. Doxycycline treatment consistently increased the rate of killing of the nematodes infected with the *tetO-YPD1* strain (*P*<0.001). These data are from a single experiment representative of three independent biological replicates. (B) *Mouse model of infection*. Kidney fungal burden measurements, percentage weight loss, and outcome score measurements of mice infected with *tetO-YPD1* cells and administered doxycycline (+DOX) or not (-DOX). Comparison of +DOX and -DOX treated groups by Kruskal-Wallis statistical analysis demonstrates significant differences for all three parameters with doxycycline treated mice giving a significantly greater outcome score (*P<0.05, ** *P*<0.01). (C) Increased virulence of cells with repressed *YPD1* expression is associated with increased numbers of fungi and inflammation in the infected kidneys. Representative images from kidneys of mice infected with *tetO-YPD1* cells and treated with (+DOX; i-iii) or without (-DOX; iv-vi) doxycycline. Sections (5 μm) were Periodic Acid-Schiff stained and post-stained with Hematoxylin. Low magnification images in (i) and (iv) (scale bar = 200 μm) show the difference in lesion number and extent of inflammatory response. Higher magnification (scale bar = 50μm) shows the presence of clusters of filamentous fungal cells (white arrows) in the lesions of doxycycline-treated mouse kidneys (ii & iii) and isolated single pseudohyphal fungal cells (white arrows) in placebo-treated mouse kidneys (v & vi). (D) Macrophage model of infection. *C*. *albicans* mediated killing of macrophages was determined by detecting the number of ruptured macrophages following co-culture with or without *tetO-YPD1* cells in the presence (+DOX) or absence (-DOX) of doxycycline. ANOVA was used to determine statistical significance (** *P* ≤ 0.01). (E) Assessment of hyphal growth following phagocytosis by *tetO-YPD1* cells in the presence (+DOX) or absence (-DOX) of doxycycline. The left panel shows that +DOX treatment resulted in a faster rate of hyphal growth. ANOVA was used to determine statistical significance (** *P* ≤ 0.01). The right panel shows images taken 61 mins post engulfment of yeast cells. The scale bar is 9 μm, and the white arrows indicate hyphal cells within the macrophage.

Initially we employed the *C*. *albicans*-*C*. *elegans* liquid medium pathogenesis assay [40] to examine the impact of *YPD1* repression on virulence. Worms were infected with *tetO-YPD1 C*. *albicans* cells which had been grown in the absence of doxycycline and thus expressing *YPD1* (Fig 2B). Subsequently, the animals were transferred to liquid medium in the presence (+DOX) or absence (-DOX) of doxycycline. Strikingly, we observed a significant increase in *C*. *elegans* killing after infection with *tetO-YPD1* cells in the presence of doxycycline in which *YPD1* expression is repressed (Fig 7A). A total of 41% of infected animals died after 72 h in liquid medium containing doxycycline compared to 11% mortality in media lacking doxycycline (*P*<0.001). Importantly, consistent with previous reports [40], doxycycline did not affect the pathogenicity of wild-type *C*. *albicans* strains towards *C*. *elegans* (S2 Fig). Hence, taken together, these results suggest that doxycycline-mediated repression of *YPD1* expression actually enhances the virulence of *C*. *albicans* towards *C*. *elegans*.

To investigate whether these results could be corroborated in a distinct infection model, we employed the three day murine intravenous challenge model of *C*. *albicans* infection [41, 42]. This model combines weight loss and kidney fungal burden measurements following 3 days of infection to give an ‘outcome score’. A higher outcome score is indicative of greater weight loss and higher fungal burdens and thus increased virulence. Twelve mice were infected intravenously with *tetO-YPD1* cells grown in medium lacking doxycycline. Following infection, one group of mice (n = 6) were orally dosed with doxycycline daily (+DOX) to repress *YPD1* expression, and the second group of placebo treated mice (n = 6) given water (-DOX). Significantly, inhibition of *YPD1* expression during infection resulted in greater weight loss, increased kidney fungal burdens, and thus higher outcome scores compared to cells which continue to express *YPD1* (Fig 7B). Consistent with previous studies [27, 39], doxycycline treatment alone does not impact on *C*. *albicans* virulence in this mouse model of systemic candidiasis (S3 Fig). Histological analysis of the kidneys revealed that there was significant inflammation associated with infection in the doxycycline treated animals (Fig 7C panel i), with little inflammation seen for the placebo treated animals (Fig 7C panel iv). Clusters of filamentous fungal cells were obvious in the doxycycline-treated mouse kidneys (Fig 7C panels ii & iii), whereas only isolated fungal cells were found in the placebo treated kidneys (Fig 7C panels v & vi). This indicates that the CFU measurements underestimate the increased fungal burden following repression of *YPD1* expression, presumably as a consequence of the increased filamentation. Nonetheless statistical analysis revealed that the difference between +DOX and −DOX cells was significant for all three parameters, including fungal burden (Fig 7B). Collectively, these studies illustrate that repression of *YPD1* expression during infection in two distinct animal models enhances the virulence of *C*. *albicans*. These results have clinical significance as they predict that a drug designed to block two-component signalling in *C*. *albicans* could actually promote the virulence of this major human fungal pathogen.

To further investigate how Ypd1 loss promotes *C*. *albicans* virulence, we employed live cell video microscopy to follow the impact of inhibition of *YPD1* expression on *C*. *albicans*-macrophage interactions. Specifically, *tetO-YPD1* cells were treated with (+DOX) or without (-DOX) doxycycline for 3 h prior to co-incubation with murine J774.1 macrophages, which were then grown in media +/-DOX, respectively. Quantitatively, there were no significant differences between the migration speed of J774.1 macrophages towards *tetO-YPD1* cells treated or not with doxycycline, or rate of engulfment of fungal cells (S4 Fig). Importantly, however, doxycycline mediated-repression of *YPD1* resulted in *C*. *albicans* cells that displayed a significantly enhanced ability to kill macrophages. It was observed that 82±4.1% of macrophages were killed following co-incubation with *tetO-YPD1* cells in the presence of doxycycline (+DOX), compared to 60±3.0% macrophages killed in the absence of doxycycline (-DOX) (*P*<0.01) (Fig 7D). The ability of *C*. *albicans* to transition to the hyphal form following phagocytosis is pivotal in triggering macrophage death [43]. Thus, the rate of hyphae formation of *tetO-YPD1* cells following phagocytosis by macrophages was measured. Hyphal growth was significantly faster in *tetO-YPD1* cells grown in the presence of doxycycline (0.35±0.032 μm/min) than in the absence of doxycycline (0.26 ± 0.023 μm/min) (Fig 7E). These results illustrate that repression of *YPD1* expression promotes hyphal growth following phagocytosis which in turn likely enhances the ability of *C*. *albicans* to kill macrophages. Given the importance of macrophages in immune responses to *C*. *albicans* infections, the increased capacity of *C*. *albicans* cells lacking Ypd1 to kill macrophages likely contributes to the enhanced virulence observed in a murine model of systemic infection.

## Discussion

**Fig 8:**
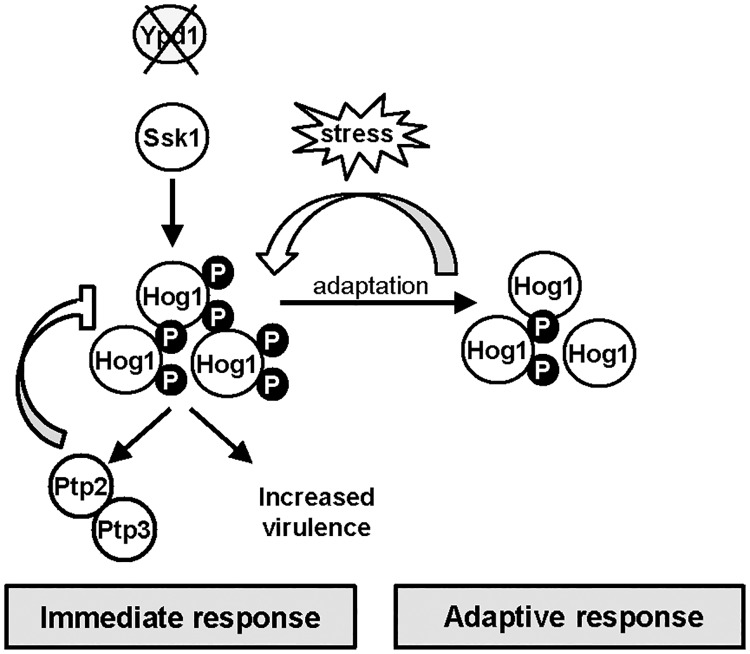
Ypd1 inactivation in *C*. *albicans* triggers Hog1 hyperactivation, increased virulence, and in the long term a reduction in Hog1 activity. Model depicting outcomes following *YPD1* loss in *C*. *albicans*. Loss of Ypd1 results in the accumulation of the unphosphorylated Ssk1 response regulator, which drives the activation of Hog1 under non-stressed conditions. The levels of Hog1 phosphorylation are modulated by the induction of the negative regulators Ptp2 and Ptp3 which allows cells to adapt and survive Ypd1 loss. Loss of Ypd1 function during infection increases the virulence of *C*. *albicans*, by possibly enhancing Hog1 activity promoting stress resistance and/or filamentation. Furthermore, *C*. *albicans* adapts to long-term activation of Hog1 by reducing the levels of the phosphorylated Hog1 kinase. This adaptation process prevents phenotypes associated with sustained SAPK activation and *ypd1Δ* cells now phenotypically resemble wild-type cells. Notably, however, this adaptation mechanism to circumvent Hog1 phosphorylation can be over-ridden following transient stress exposure and thus sustained Hog1 activation is restored.

The generation of fungal pathogen-specific drugs is hindered by the conservation of many potential drug-targets in the human host. Thus the complete absence of two-component related proteins in metazoans, but their presence in fungi, has rendered such pathways attractive drug-targets [22, 44, 45]. In *S*. *cerevisiae*, and the human fungal pathogen *C*. *neoformans*, loss of the two-component phosphorelay protein Ypd1 causes lethality due to the sustained activation of their respective SAPK pathways [20, 21]. However, as reported during the course of this work [25], a major human fungal pathogen, *C*. *albicans*, can tolerate sustained activation of the Hog1 SAPK pathway triggered by loss of Ypd1. We have significantly advanced our understanding of this observation in three main ways. Firstly we find that the constitutive activation of Hog1 in cells lacking *YPD1* is mediated through two-component mediated regulation of the Ssk1 response regulator, and that the pleiotropic phenotypes associated with Ypd1 loss are dependent on Ssk1-mediated Hog1 activation. Secondly, we have provided novel insight into the mechanisms by which *C*. *albicans* survives and adapts to sustained SAPK activation. Thirdly, we show that inactivation of *YPD1* promotes, rather than reduces, the virulence of *C*. *albicans*, and we provide evidence to suggest that this is mediated at least in part through effects on fungus-phagocyte interactions. A model summarising the major findings from this work is depicted in Fig 8.

One question raised by this study is why is sustained SAPK activation tolerated in some fungi but not others? In *S*. *cerevisiae*, prolonged SAPK activation is reported to lead to cell death by causing an increase in reactive oxygen species (ROS) thus triggering apoptosis [12]. Although we do not know whether sustained Hog1 activation triggers an increase in intracellular ROS in *C*. *albicans*, it is worth noting that *C*. *albicans* is considerably more resistant to oxidative stress than *S*. *cerevisiae* [46]. In *C*. *neoformans*, lethality due to Hog1 hyperactivation is attributed to the over-accumulation of intracellular glycerol [47]. In *C*. *albicans*, sustained Hog1 activation in *ypd1Δ* cells also triggers an increase in intracellular glycerol levels, presumably contributing to the associated swollen cell phenotype. Interestingly, this is tolerated in these mutant cells and indeed it is noteworthy that *C*. *albicans* can withstand higher levels of osmotic stress (which triggers glycerol biosynthesis) than many other fungal species [46]. However, perhaps a key factor in maintaining the viability of *ypd1Δ* cells, is the significant induction of the tyrosine phosphatase encoding genes *PTP2* and *PTP3* which negatively regulate Hog1 phosphorylation [35]. Interestingly, the lethality associated with *ypd1Δ*-mediated sustained Hog1 activation in *S*. *cerevisiae* can be by-passed by artificial over-expression of either *PTP2* or *PTP3* [31, 32], suggesting that *C*. *albicans* actually adopts this strategy to prevent catastrophic levels of Hog1 activation. Consistent with this hypothesis is the observation that treatment of *C*. *albicans ypd1Δ* cells with the tyrosine phosphatase inhibitor, arsenite, triggers a significant increase in levels of Hog1 phosphorylation and cell death. Furthermore, deletion of *PTP3* together with deletion of one copy of *PTP2*, triggers dramatic increases in the level of phosphorylated Hog1 in *tetO-YPD1* cells and furthermore, virtually abolishes cell growth following doxycycline-mediated repression of *YPD1*. It is not clear why we could not generate *tetO-YPD1* cells lacking both *PTP3* and *PTP2*, as a double *ptp3Δ ptp2Δ* mutant has previously been characterised [35], although it is possible that basal *YPD1* expression levels are altered in the *tetO-YPD1* background. Notably, however, we find that the basal level of expression of *PTP2* plays a major role in preventing Hog1 activation under non-stress conditions, as significant increases in the levels of Hog1 phosphorylation are seen upon deleting one copy of *PTP2*. Similar findings have been reported in both *S*. *cerevisiae* and *C*. *neoformans* where basal levels of Hog1 are significantly increased in *ptp2*Δ mutant cells [31, 34]. Taken together, the data presented in this paper strongly support the model that induction of *PTP3* and *PTP2* expression, together with the basal expression of *PTP2*, allow *C*. *albicans* to survive loss of the Ypd1 phosphorelay protein.

The viability of *C*. *albicans* cells lacking Ypd1 has allowed an investigation of how fungal cells adapt to long-term sustained SAPK activation. Remarkably, we find that cells evoke a mechanism that prevents the long-term constitutive phosphorylation of Hog1. Moreover, this decrease in Hog1 phosphorylation is actually accompanied by a reduction in the levels of *PTP2* and *PTP3*, the negative regulators of Hog1, raising the possibility that an upstream signalling branch to Hog1 is inhibited instead. Furthermore, this mechanism is reversible as it can be over-ridden following a transient exposure to stress that requires Hog1 activity. Do these observations that *C*. *albicans* can survive and adapt to sustained SAPK activation have physiological relevance? As a human commensal, this fungal pathogen is continuously exposed to host-imposed stresses. Indeed, Hog1 is vital for *C*. *albicans* to exist commensally in the gut [11], cause systemic infections [8, 10], and survive phagocytosis [9]. Thus, the ability of this pathogen to adapt to sustained SAPK activation by actively modulating Hog1 phosphorylation levels may be important to promote survival in certain host niches that continuously generate a stressful environment. Furthermore, we propose that the capacity of *C*. *albicans* to restore levels of SAPK phosphorylation, upon subsequent exposure to conditions that require Hog1-mediated stress responses, underpins the flexibility needed to allow adaptation of *C*. *albicans* to the range of environments encountered within the host [48]. Interestingly, long-term SAPK activation can be tolerated in other organisms. For example, in some human cell types sustained SAPK activation promotes either cell survival [49] or cellular differentiation [50], rather than apoptosis. However, whether similar mechanisms are evoked in humans and *C*. *albicans*, to adapt to sustained SAPK activation is unknown.

The main findings reported here in this paper focused on cells in which *HOG1* is expressed from its native chromosomal locus. However, during the course of this work we noted that the cellular response of *C*. *albicans* to *YPD1* loss differed depending on whether the *HOG1* gene was present at its native locus, or re-integrated at the *RPS10* locus in *hog1Δ ypd1Δ+HOG1* cells. In both instances, cells adapted to long term SAPK activation by reducing the levels of phosphorylated Hog1 (S5A and S5B Fig). However, when *HOG1* was reintegrated at the *RPS10* locus, this was also accompanied by a reduction in *HOG1* mRNA levels (S5C Fig). Consistent with the decrease in Hog1 levels, *hog1Δ ypd1Δ+HOG1* cells changed dramatically over time to phenocopy *hog1Δ* cells (S5D Fig). These results contrast significantly with those from *ypd1Δ* cells when *HOG1* is expressed from its normal locus where the cells change over time to phenocopy wild type cells (compare Fig 5 and S5 Fig). Indeed, the *ypd1Δ*-dependent down-regulation of *HOG1* located at the *RPS10* locus was independent of *HOG1* promoter and terminator sequences (S5E Fig), suggesting that this is a genome position-effect rather than a gene-specific phenomenon. Insertion of genes at the *RPS10* locus has been employed by numerous labs to successfully generate re-integrant strains [51, 52]. Significantly, the present study has highlighted the importance of studying a gene at its native chromosomal locus when investigating the role and/or regulation of the gene.

Two-component proteins represent attractive antifungal targets as these signal transduction proteins are absent in metazoans [22, 44, 45]. Indeed, deletion of any one of the three *C*. *albicans* histidine kinases, or the Ssk1 response regulator, attenuate virulence in mouse systemic infection models. Furthermore, the Ypd1 phosphorelay protein has generated significant interest as an antifungal target, due to the lethality associated with its deletion in both *S*. *cerevisiae* and the human fungal pathogen *C*. *neoformans* [20, 21]. However, in this study we report that inhibition of *YPD1* expression during infection actually increases the virulence of *C*. *albicans* in two distinct infection models; a *C*.*elegans* pathogenesis model [40] and a murine model of systemic candidiasis [41, 42]. Histology images from murine kidneys show that, similar to that seen *in vitro*, repression of *YPD1* during infection results in clusters of highly filamentous cells. Enhanced filamentation *in vivo* has previously been shown to increase virulence [39], and thus the increased virulence associated with *YPD1* loss *in vivo* may be due to the formation of clusters of hyper-filamentous cells. Moreover, we show that drug mediated inhibition of *YPD1* expression increases the efficacy by which *C*. *albicans* can kill macrophages. This is likely related to the observation that Ypd1 loss also results in increased filamentation within this infection model, as hyphae formation within the macrophage promotes *C*. *albicans*-mediated killing of macrophages by triggering pyroptosis [53], and by mechanically rupturing the macrophage cell membrane [54].

The molecular basis underlying the increased virulence seen upon repressing *YPD1* expression is unknown, but given that Hog1 is essential for *C*. *albicans* virulence in several infection models [8–11], it is possible that the concomitant increases in Hog1 activity as a consequence of Ypd1 loss promotes *C*. *albicans* survival in the host (Fig 8). It is important to note here that the filamentous phenotype exhibited by cells lacking Ypd1 is due to the concomitant sustained Hog1 activation. To explore whether the enhanced virulence seen upon Ypd1 loss was dependent on Hog1, we compared the virulence of wild-type, *hog1Δ* and *hog1Δ ypd1Δ C*. *albicans* cells in the three day murine infection model described above. Although it was not feasible to analyse *ypd1Δ* cells in this model due to the highly flocculent nature of this strain, *hog1Δ* and *hog1Δ ypd1Δ* cells demonstrated equally impaired virulence in the three-day murine systemic infection model (S1 Table). Thus the impaired virulence exhibited by *hog1Δ* cells is not improved upon deletion of *YPD1* and, therefore, is consistent with the hypothesis that the enhanced virulence observed upon loss of *YPD1* may be dependent on the concurrent increased activation of the Hog1 SAPK to promote fungal stress resistance and/or filamentation.

Importantly, the data presented here illustrating that Ypd1 loss potentiates *C*. *albicans* virulence, indicates that antifungals administered to treat *C*. *albicans* infections that target Ypd1 have the potential to actually enhance the virulence of this major pathogen. It is also noteworthy that SAPKs are important for the virulence of other human and plant fungal pathogens [55]. Consequently, any compound capable of inhibiting two-component signalling that leads to SAPK activation, may promote the survival of other fungal pathogens when encountering host-imposed stresses. Thus, in conclusion, our findings question the validity of the Ypd1 protein as a broad-spectrum antifungal target. Indeed, the significant differences in stress-signalling outputs between *S*. *cerevisiae* and *C*. *albicans*, underscores the importance of directly studying predicted ‘essential’ genes in pathogenic fungi, rather than in model yeast.

## Materials and methods

### Strains and growth media

All the *C*. *albicans* strains used in this study are listed in Table 1. Cells were grown at 30°C in YPD rich medium [56]. Addition of 20μg/ml doxycycline was used to repress expression from the *tetO* promoter.

**Table 1:** Strains used in this study.

Strain	Relevant Genotype	Source
THE1	*ade2*::*hisG/ade2*::*hisG*, *ura3*::*imm434/ura3*::*imm434*, *ENO1/eno1*::*ENO1-tetR-ScHAP4AD-3×HA-ADE2*	[27]
JC1586	THE1 *ypd1*::*hisG/ypd1*::*99t-YPD1-URA3*	This study
JC2188	THE1 *ypd1*::*hisG/ypd1*::*99t-YPD1-URA3*, *ptp3*::*loxP/ptp3*::*loxP*	This study
JC2195	THE1 *ypd1*::*hisG/ypd1*::*99t-YPD1-URA3*, *ptp3*::*loxP/ptp3*::*loxP PTP2/ptp2*::*loxP*	This study
RM1000	*ura3*:: *imm434/ura3*::*imm434*, *his1*::*hisG/his1*::*hisG*	[62]
JC21	RM1000 + CIp20	[6]
JC50	RM1000 *hog1*::*loxP-ura3-loxP*, *hog1*::*loxP-HIS1-loxP* + CIp20	[6]
JC52	RM1000 *hog1*::*loxP-ura3-loxP*, *hog1*::*loxP-HIS1-loxP* + CIp20*-HOG1*	[6]
JC1475	RM1000 *hog1*::*loxP-ura3-loxP*, *hog1*::*loxP-HIS1-loxP*, *ypd1*::*hisG/ypd1*::*hisG* + CIp20	This study
JC1478	RM1000 *hog1*::*loxP-ura3-loxP*, *hog1*::*loxP-HIS1-loxP*, *ypd1*::*hisG/ ypd1*::*hisG* + CIp20*-HOG1*	This study
JC1850	RM1000 *hog1*::*loxP-ura3-loxP*, *hog1*::*loxP-HIS1-loxP pACT-HOG1*	This study
JC1851	RM1000 *hog1*::*loxP-ura3-loxP*, *hog1*::*loxP-HIS1-loxP*, *ypd1*::*hisG/ypd1*::*hisG* pACT1*-HOG1*	This study
JC1859	RM1000 *hog1*::*loxP-ura3-loxP*, *hog1*::*loxP-HIS1-loxP*, *ypd1*::*hisG/ypd1*::*hisG* CIp20-*HOG1*	This study
JC1860	RM1000 *hog1*::*loxP-ura3-loxP*, *hog1*::*loxP-HIS1-loxP*, *ypd1*::*hisG/ypd1*::*hisG* CIp20-*HOG1*	This study
JC1990	RM1000 *ypd1*::*HIS1/ypd1*::*hisG-URA3-hisG*	This study
JC2001	RM1000 *ypd1*::*HIS1/ypd1*::*hisG* CIp20	This study
JC2002	RM1000 *ypd1*::*HIS1/ypd1*::*hisG* CIp20-*YPD1*	This study
SN148	*arg4Δ*/*arg4*Δ *leu2Δ*/*leu2Δ his1Δ*/*his1Δ*, *ura3Δ*::*imm434*/ *ura3Δ*::*imm434*, *iro1Δ*::*imm434/ iro1Δ*::*imm434*	[63]
JC1552	SN148 *ssk1*::*loxP-ARG4 -loxP/ssk1*::*loxP-HIS1-loxP* + CIp20	[29]
JC1683	SN148 *ssk1*::*loxP-ARG4 -loxP/ssk1*::*loxP-HIS1-loxP*, *ypd1*::*hisG/ ypd1*::*hisG* + CIp20	This study
JC1704	SN148 *ssk1*::*loxP-ARG4 -loxP/ssk1*::*loxP-HIS1-loxP*, *ypd1*::*hisG/ ypd1*::*hisG* + CIp20-*SSK1*	This study
JC112	BWP17 *pbs2*::*loxP-HIS1-loxP/ PBS2-HM*:*URA3*	[64]
JC124	BWP17 *pbs2*::*loxP-HIS1-loxP/ PBS2*^*DD*^*-HM*:*URA3*	[10]
JC126	BWP17 *pbs2*::*loxP-HIS1-loxP/ PBS2*^*AA*^*-HM*:*URA3*	[10]

### Strain construction

The strains used in this study are listed in Table 1, and oligonucleotides used for their construction are listed in S2 Table.

#### Doxycycline-mediated repression of YPD1

To place *YPD1* under control of the *tetO* promoter, one allele of *YPD1* was disrupted in strain THE1 [27] to generate JC1420. Ura-blasting was used to delete one *YPD1* allele [57] using a *ypd1*::*hisG-URA3-hisG* cassette generated by amplifying regions flanking the *YPD1* open reading frame (ORF), using the oligonucleotide primer pairs Ypd1BglIIF/Ypd1BglIIR and Ypd1BamHIF/Ypd1BamHIR respectively, and sequentially ligating into p5921. The *ypd1*::*hisG-URA3-hisG* disruption cassette deleted the entire 184 codon *YPD1* ORF. The strain was then passed over 5FOA to recycle the *URA3* marker. To place *YPD1* under the *tetO* promoter, sequences containing nucleotides −382 to −71 (sequence A) and −8 to +294 (sequence B) relative to the start codon of *YPD1* were PCR amplified using the primer pairs YPD1KPNIA.F/YPD1CLAIA.R and YPD1SACIIB.F/YPD1SACIIB.R respectively. The PCR products were digested with *Kpn*I/*Cla*I (sequence A) and *Sac*II (sequence B) and sequentially ligated into the p99CAU1 plasmid [27]. The 2.8kb *URA3-TET-YPD1* construct was then released from the plasmid as a *Kpn*I-*Sac*II fragment and introduced into JC1420 cells to generate strain *tetO*-*YPD1* (JC1586). All gene disruptions, and correct integration of fragments, were confirmed by PCR.

#### Deletion of YPD1

To delete *YPD1*, a disruption cassette comprising of the *HIS1* gene flanked by *loxP* sites and 80 base pairs of DNA sequence corresponding to regions 5’ and 3’ of the *YPD1* ORF, were generated by PCR using the oligonucleotide primers YPDdelF and YPDdelR and the plasmid template pLHL2 respectively. The disruption cassette was transformed into *C*. *albicans* RM1000 to disrupt one allele of *YPD1* and generate strain JC1338. The second copy of *YPD1* was deleted using the ura blasting cassette as described above, to generate JC1990. To re-introduce *YPD1*, the *YPD1* locus was PCR amplified using the oligonucleotide primers Ypd1PromF and Ypd1TermR and cloned into the integrating plasmid CIp20 [51]. CIp20 and CIp20-YPD1 were integrated at the *RPS10* locus in a 5-FOA resistant derivative of JC1990 to create strains JC2001 and JC2002 respectively. Both copies of *YPD1* were also deleted in *hog1Δ* cells (JC47; [6]) and *ssk1Δ* cells (JC1402; [29]) using the ura-blaster cassette to generate strains JC1450 (*hog1Δ ypd1Δ*) and JC1656 (*ssk1Δ ypd1Δ*), respectively. Single copies of either *HOG1* or *SSK1* were reintegrated into these strains. The *SSK1* gene plus 970bp of the promoter region and 237bp of the terminator region was amplified by PCR, using the oligonucleotide primers Ssk1BamHIF and Ssk1BamHIR, and ligated into the *Bam*HI site of CIp20 [51] to generate CIp20-*SSK1*. CIp20-*HOG1* [6] and CIp20-*SSK1* were digested with *Stu*I and integrated at the *RPS10* locus in the *hog1Δ ypd1Δ* and *ssk1Δypd1Δ* double mutants, respectively, to generate strains JC1478 and JC1704. The CIp20 vector was also integrated at the *RPS10* locus in the *ssk1Δ ypd1Δ* and *hog1Δ ypd1Δ* double mutants to generate strains JC1683 and JC1475 respectively.

#### Deletion of PTP3 and PTP2

These mutants were constructed using the Clox system with nourseothricin selection [52]. To create the *ptp3Δ* mutant, the NAT1-Clox cassette was PCR amplified using primer pairs PTP3delF and PTP3delR, and introduced into *tetO-YPD1* cells to sequentially delete both *PTP3* alleles and generate strain (JC2188). The same strategy was used to delete *PTP2* in JC2188 using the primer pairs PTP2delF and PTP2delR. One copy of *PTP2* was deleted generating the strain *tetO-YPD1 ptp3Δ ptp2/PTP2* (JC2195). Despite extensive efforts the second copy of *PTP2* could not be deleted in JC2195.

#### Ectopic expression of HOG1

The *HOG1* open reading frame was amplified by PCR, using the primer pair Hog1HindIIIF and Hog1HindIIIR and CIp20-*HOG1* as template, and ligated into the *Hin*dIII site between the *CaACT1* promoter and the *ScCYC1* terminator in pACT1 [58]. The resulting pACT1-*HOG1* plasmid was integrated at the *RPS10* locus in *hog1Δ ypd1Δ* cells, and two individual transformants (JC1850, JC1851) were immediately frozen at -80°C to prevent reversion of *ypd1Δ*-dependent phenotypes. The CIp20-*HOG1* plasmid [6], which contains the *HOG1* ORF and promoter and terminator sequences, was integrated at the *RPS10* locus in the *hog1Δ ypd1Δ* cells, and two individual transformants (JC1859, JC1860) also immediately frozen at -80°C. Correct integration at the *RPS10* locus and the DNA sequences of the integrated open reading frames were confirmed by PCR and DNA sequencing, respectively.

### Glycerol assay

Glycerol concentrations were determined using The Free Glycerol Determination Kit (Sigma-Aldrich), following the manufacturer’s instructions. Three independent biological replicates were performed.

### Hog1 phosphorylation assays

Protein extracts were prepared from mid-exponential phase cells and phosphorylated Hog1 was detected by western blot analysis with an anti-phospho-p38 antibody (New England Biolabs) as described previously [6]. Blots were stripped and total levels of Hog1 were determined by probing with an anti-Hog1 antibody (Santa Cruz Biotechnology), and in some cases protein loading determined using an anti-tubulin antibody (DSHB, University of Iowa).

### Northern blot analysis

RNA preparation and Northern blot analyses were performed as described previously [6]. Gene-specific probes were amplified by PCR from genomic DNA using oligonucleotide primers specific for *YPD1*, *GPD2*, *RHR2*, *PTP2*, *PTP3*, *HOG1* and *ACT1* (S1 Table).

### Stress sensitivity tests

*C*. *albicans* strains were grown at 30°C to exponential phase and then 10 fold serial dilutions were spotted onto YPD plates containing the indicated compounds. Plates were incubated at 30°C for 24 h.

### Microscopy

Differential interference contrast images were captured using a Zeiss Axioscope microscope as described previously [6].

### Virulence analysis

#### C. elegans pathogenesis assay

This was performed essentially as described previously [40]. Freshly grown *C*. *albicans* cells were inoculated into 10 ml of YPD and grown overnight at 30°C. The following day cells were diluted to an OD_660_ 0.2, and 10 μl of yeast was spotted onto a 5-cm plate containing Brain Heart Infusion BHI agar and kanamycin (45 μg/ml), and incubated overnight at room temperature. Synchronized adult *C*. *elegans* (*glp-4*) nematodes grown at 25°C were washed from NGM-L plates containing their normal food source (*E*. *coli* OP50) using sterile M9 buffer. Approximately 400 washed animals were then pipetted to the centre of the *C*. *albicans* lawn and incubated at 25°C for 4 h. Worms were then transferred to sterile M9 medium and washed three times. At least 60 worms were then pipetted into a single well of a six-well tissue culture plate containing 2 ml of liquid medium (80% M9, 20% BHI) supplemented or not with doxycycline (20μg/ml). The culture plate was incubated at 25°C, and worm survival was scored daily under a stereomicroscope. Worms were considered to be dead if they did not move in response to probing with a pick, and were removed from the assay. Survival was analysed using the Kaplan-Meier method and differences in *C*. *elegans* survival were determined by the log-rank test.

#### Murine intravenous challenge assay

Murine BALB/c female mice (6–8 weeks old, Envigo UK) were housed in randomly assigned groups of 6, with food and water provided *ad libitum*. In the first set of experiments mice were infected intravenously via a lateral tail vein with 4.5e4 CFU/g of *C*. *albicans tetO-YPD1* cells grown in medium lacking doxycycline. Treatments were randomly assigned to groups of infected mice. One group of mice (n = 6) was gavaged (orally dosed) with 2 mg doxycycline daily (+DOX), and the second group of placebo treated mice (n = 6) orally dosed with water (-DOX). In the second group of experiments, mice (n = 6) were infected with 2.5e4 CFU/g wild-type (JC21), *hog1Δ* (JC50) or *hog1ypd1Δ* (JC1475) cells. Body weights were recorded daily. 72 h after challenge the animals were weighed, humanely terminated and kidneys removed aseptically. Fungal burdens were measured by viable counts for two half kidneys per animal. Virulence was assessed by fungal kidney burdens at 72 h, and by percent weight change over 72 h, from which an outcome score was calculated [41, 42]. The wild-type (JC21), *hog1Δ* (JC50) and *hog1ypd1Δ* (JC1475) cells contain a single copy of *URA3* integrated at the *RPS10* locus and thus the levels of *URA3* expression within these strains should be identical. In addition, the *tetO-YPD1* strain is Ura3^+^ (Table 1), and the only difference between the two groups of mice injected with this strain is doxycycline treatment. Hence the levels of *URA3* expression in *tetO-YPD1* cells in both -DOX and +DOX groups of mice should be identical. This is important to avoid any indirect effects on virulence due to differing levels of *URA3* expression [59]. Furthermore, as previously reported, doxycycline treatment alone does not impact on *C*. *albicans* virulence (S3 Fig). Differences were tested by Kruskal-Wallis statistical analysis using IBM SPSS Statistics v24.

Kidney sections were prepared from formalin-fixed halves of kidneys from infected mice. Kidney halves were embedded in paraffin wax and 5 μm sections cut. Sections were deparaffinized and underwent Periodic Acid-Schiff staining, with hematoxylin post-staining. Images were captured using a Zeiss Axioscan Z1 slide scanner.

#### Phagocytosis assays and live-cell video microscopy

These experiments were performed as described previously [60]. The J774.1 mouse macrophage cell line (European Collection of Cell Culture) was maintained at 37°C and 5% CO_2_ in Dulbecco`s modified Eagle`s medium (DMEM) (Lonza, Slough, UK) supplemented with 10% heat-activated fetal calf serum (FCS) (Biosera, Ringmer, United Kingdom). Cells were seeded at a density of 1.2 x10^5^ cells/well in an 8-well slide and incubated overnight at 37°C and 5% CO_2_. Prior to co-incubation growth medium in wells was replaced with prewarmed CO_2_-independent medium containing 10% FCS (Invitrogen) and supplemented or not with doxycycline (20 μg/ml). Overnight cultures of the *tetO-YPD1* strain grown in rich media lacking doxycycline (20 μg/ml) was diluted into fresh media supplemented or not with doxycycline (20 μg/ml) and incubated for 3 h, following which cells were washed in sterile phosphate-buffered saline (PBS) (pH7.4) and co-cultured with J774.1 cells at a MOI of 3:1 respectively. DIC images of co-cultured cells were taken at 1-min intervals over a 6 h time course using an Ultra VIEW VoX spinning-disk microscope (Nikon, Surrey, United Kingdom) and an EMCCD camera. Two independent experiments were carried out and 6 movies analysed from each experiment per *C*. *albicans* strain. Fifty macrophages were randomly selected from each movie and phagocytic activity determined. Volocity 5.0 imaging software (Improvision, PerkinElmer, Coventry, United Kingdom) was used for data acquisition and analysis as described previously [61]. The software enabled the determination of the engulfment time, uptake events, rate of *C*. *albicans* filamentation, and macrophage survival. Macrophage survival/killing was determined by detecting the number of ruptured macrophages within sample populations of 50. Differences were tested for statistical significance by one-way analysis of variance (ANOVA) with Bonferonni`s post hoc comparisons.

### Ethics statement

All animal experiments were conducted in compliance with United Kingdom Home Office licenses for research on animals (project license number PPL 60/4135), and were approved by the University of Aberdeen Animal Welfare and Ethical Review Body (AWERB). Animal experiments were minimised, and all animal experimentation was performed using approaches that minimised animal suffering and maximised our concordance with the 3Rs.

## Supporting information

S1 Table Effect of deleting Hog1, or Hog1 and Ypd1, on experimental infection outcome. Infection with either *hog1Δ* or *hog1Δ ypd1Δ* cells resulted in weight increases, lower kidney fungal burdens and, thus, lower outcome scores compared to that observed with wild-type cells. Statistical analysis revealed that for all parameters; weight loss, kidney fungal burden and outcome score, the difference between each mutant strain with wild-type cells was significant (*P*<0.05). However, there was no significant difference for any of the parameters between *hog1Δ* and *hog1Δ ypd1Δ* cells.
(DOCX)

S2 Table Oligonucleotides used in this study. (DOCX)

S1 Fig Doxycycline does not stimulate Hog1 phosphorylation. The indicated strains were treated or not with 20μg/ml doxycycline (DOX) for 1 h, and cell extracts were analysed for phosphorylated Hog1 by western blotting. Blots were probed for phosphorylated Hog1 (Hog1-P), stripped and reprobed for total Hog1 (Hog1). Hog1 phosphorylation is stimulated upon doxycycline-mediated reduction of *YPD1* expression in *tetO-YPD1* cells (JC1586), but not in the parental THE1 and THE1 *ypd1*::*hisG* (JC1420) strains following doxycycline treatment.
(TIFF)

S2 Fig Doxycycline treatment does not affect *C*. *albicans* virulence in a *C*. *elegans* model of infection. Nematodes were infected with wild-type THE1 or wild-type JC806 cells and transferred to liquid medium either with (+DOX) or without (-DOX) doxycycline. Doxycycline had no significant impact on nematode killing infected with either wild-type strain in (*P*>0.05). These data are from a single experiment representative of two independent biological replicates.
(TIF)

S3 Fig Doxycycline treatment does not affect *C*. *albicans* virulence in a murine infection model. Kidney fungal burden measurements, percentage weight loss, and outcome score measurements of mice infected with wild-type *C*. *albicans* cells (SC5314) and administered doxycycline (+DOX) or not (-DOX). Comparison of +DOX and -DOX treated groups by Kruskal-Wallis statistical analysis found no significant differences for any of the three parameters.
(TIF)

S4 Fig Doxycycline treatment does not affect rate of uptake of *C*. *albicans tetO-YPD1* cells. (A) Percentage uptake of *tetOYPD1* cells grown in the presence (+DOX) or absence (-DOX) of doxycycline. No significant difference between uptake events + or − minus Dox by J774.1 macrophages after 6h co incubation was detected. (B) Engulfment time required for the ingestion of *tetOYPD1* cells grown in the presence (+DOX) or absence (-DOX) of doxycycline. The bars represent the average time (minutes) taken for the complete engulfment of the cells by J774.1 macrophages. No significant differences between the rate of engulfment of fungal cells − or + Dox treatment were detected.
(TIF)

S5 Fig *HOG1* is regulated differently at the *RPS10* locus in response to sustained Hog1 activation. (A) Hog1 phosphorylation is not sustained in *hog1Δypd1Δ+HOG1* cells over time and this is accompanied by a reduction in total Hog1 protein levels. Western blot analysis of whole cell extracts isolated from exponentially growing *hog1Δ+HOG1* (JC52) and *hog1Δypd1Δ+HOG1* (JC1478) cells taken from rich media plates after the number of days indicated. *indicates a non-specific band. (B) Comparison of Hog1 phosphorylation and Hog1 levels in *hog1Δypd1Δ+HOG1* and *ypd1Δ* (JC2001) cells. Western blots were also probed for tubulin (Tub) in addition to phosphorylated (Hog1-P) and total Hog1 (Hog1). (C) *GPD2* expression is not sustained in *hog1Δypd1Δ+HOG1* cells and this correlates with a decline in *HOG1* mRNA levels. Northern blot analysis of *HOG1* and *GPD2* expression in exponentially growing cells taken from rich media plates after the number of days indicated. The relative expression of *HOG1* and *GPD2* to the *ACT1* loading control in is shown. (D) *hog1Δ ypd1Δ+HOG1* cells gradually accumulate phenotypes characteristic of *hog1Δ* cells. Approximately 10^4^ cells, and 10-fold dilutions thereof, of exponentially growing cells taken from rich media plates after Day 1 or Day 9 were spotted onto plates containing; NaAsO_2_ (1.5 mM), calcofluor white (30 μg/ml) and NaCl (0.5 M). Plates were incubated at 30°C for 24 hrs. Micrographs illustrating the morphology of *hog1Δ+HOG1* and *hog1Δypd1Δ+HOG1* cells at Day 1 and Day 9 are also shown. (E) Reduction of Hog1 levels at the *RPS10* locus occurs independently of the *HOG1* promoter sequence. Western blot analysis of whole cell extracts isolated from two freshly isolated independent *hog1Δ ypd1Δ* strains expressing *HOG1* integrated at the *RPS10* locus from its native promoter (JC1859, JC1860; left panel) or the *ACT1* promoter ACT1p-*HOG1* (JC1850, JC1851; right panel). Blots were processed as described in B.
(TIF)
