# Supplementary material for: Blocking two-component signalling enhances Candida albicans virulence and reveals adaptive mechanisms that counteract sustained SAPK activation
Source: PLoS Pathog. 2017 Jan 30;13(1):e1006131. doi: 10.1371/journal.ppat.1006131 (PMC5300278; doi:10.1371/journal.ppat.1006131)
Supplement: S1 Table — Infection with either hog1Δ or hog1Δ ypd1Δ cells resulted in weight increases, lower kidney fungal burdens and, thus, lower outcome scores compared to that observed with wild-type cells. Statistical analysis revealed that for all parameters; weight loss, kidney fungal burden and outcome score, the difference between each mutant strain with wild-type cells was significant (P<0.05). However, there was no significant difference for any of the parameters between hog1Δ and hog1Δ ypd1Δ cells. (DOCX) [file ppat.1006131.s001.docx]

| **Strain** | **Kidney Burden**  **(log 10 CFU/g)** | **% Weight Change** | **Outcome Score** |
| --- | --- | --- | --- |
| *Wt*  (JC26) | 4.4 ± 0.4 | -1.2 ± 3.6 | 5 ± 2.0 |
| *hog1Δ*  (JC50) | 2.9 ± 0.3 | 3.3 ± 3.5 | 1.2 ± 1.8 |
| *hog1Δypd1Δ*  (JC1425) | 2.3 ± 0.5 | 2.4 ± 1.6 | 1.1 ± 1.2 |

**Table S1. Effect of deleting Hog1, or Hog1 and Ypd1, on experimental infection outcome.**
