# Supplementary material for: Blocking two-component signalling enhances Candida albicans virulence and reveals adaptive mechanisms that counteract sustained SAPK activation
Source: PLoS Pathog. 2017 Jan 30;13(1):e1006131. doi: 10.1371/journal.ppat.1006131 (PMC5300278; doi:10.1371/journal.ppat.1006131)
Supplement: S2 Table — (DOCX) [file ppat.1006131.s002.docx]

| **Table S2. Oligonucleotides used in this study**. |  |  |
| --- | --- | --- |

| Oligonucleotide | Sequence 5’–3’ |
| --- | --- |
| Ypd1BglIIF | aggaagatctcctgtaccaatagaaaactc |
| Ypd1BglIIR | aggaagatctgtttcaagtttcctcttag |
| Ypd1BamHIF | gcgcggatcccttattataagaatgccac |
| Ypd1BamHIR | gcgcggatccaaatgagaagcatagcac |
| YPD1KPNIA.F | ccggggtacccctgtaccaatagaaaac |
| YPD1CLAIA.R | ggccatcgatgccgtcttgattggttatc |
| YPD1SACIIB.F | ttccccgcggctttcataatgtcagaag |
| YPD1SACIIB.R  Hog1HindIIIF  Hog1HindIIIR | ttccccgcgggttgatcttatggccatag  ggcccaagcttatgtctgcagatggagaatttac  cggcccaagcttttaagctccgttggcggaatc |
| Ssk1BamH1F  Ssk1BamH1R  PTP2delF  PTP2delR  PTP3delF  PTP3delR  YPD1F | gcgcggatccctacgggacaacacgttatg  gcgcggatccggtgttcttggtatgatccg  ttacttccccacttctgttttcacc**atg**tcaactgttgaaaattctaatactacatttcatcaccctacttat  caacaacaacaacaacaacaatctagtacggccagtgaattgtaata  aattttataaaaatttcataacaagcaataaattgattataattttgtaccattgaaattctttgttttctttga  tgttgaatcaatttataaatcaaattcggaattaaccctcactaa  ttgacgagctgctgccaaccaacaaagtggaaggttgaaacgaaacgagaaaattaaaaaaaa  cacacaacaaaagttcagtcccacaccagtaatagttacggccagtgaattgtaata  tttttgcaataataatccaataaagcttcataaatcatataatattgtgttaatgtctgaaccattgatattc  tcaattttcgataatgattagtgattttcggaattaaccctcactaa  atgtcagaagataaattacaaaaattac |
| YPD1R | gtcatcgcttgattcatccgg |
| HOG1F | ctgcagatggagaatttacaagaacc |
| HOG1R | tggagaattgatgcacgtgatctttccc |
| ACT1F | gatgaagcccaatccaaaag |
| ACT1R | ggagttgaaagtggtttggt |
| GPD2F | tgtattgtcggttccggtaactgg |
| GPD2F  PTP2F  PTP2R  PTP3F  PTP3R | tgtattgtcggttccggtaactgg  atgtcaactgttgaaaattctaa  tgaattccgttttaataatgttg  atgacattcacattccctaata  ttggttcacttggtttgaatg |
|  |  |
|  |  |
