# Supplementary figures and images for: Blocking two-component signalling enhances Candida albicans virulence and reveals adaptive mechanisms that counteract sustained SAPK activation
Source: PLoS Pathog. 2017 Jan 30;13(1):e1006131. doi: 10.1371/journal.ppat.1006131 (PMC5300278; doi:10.1371/journal.ppat.1006131)

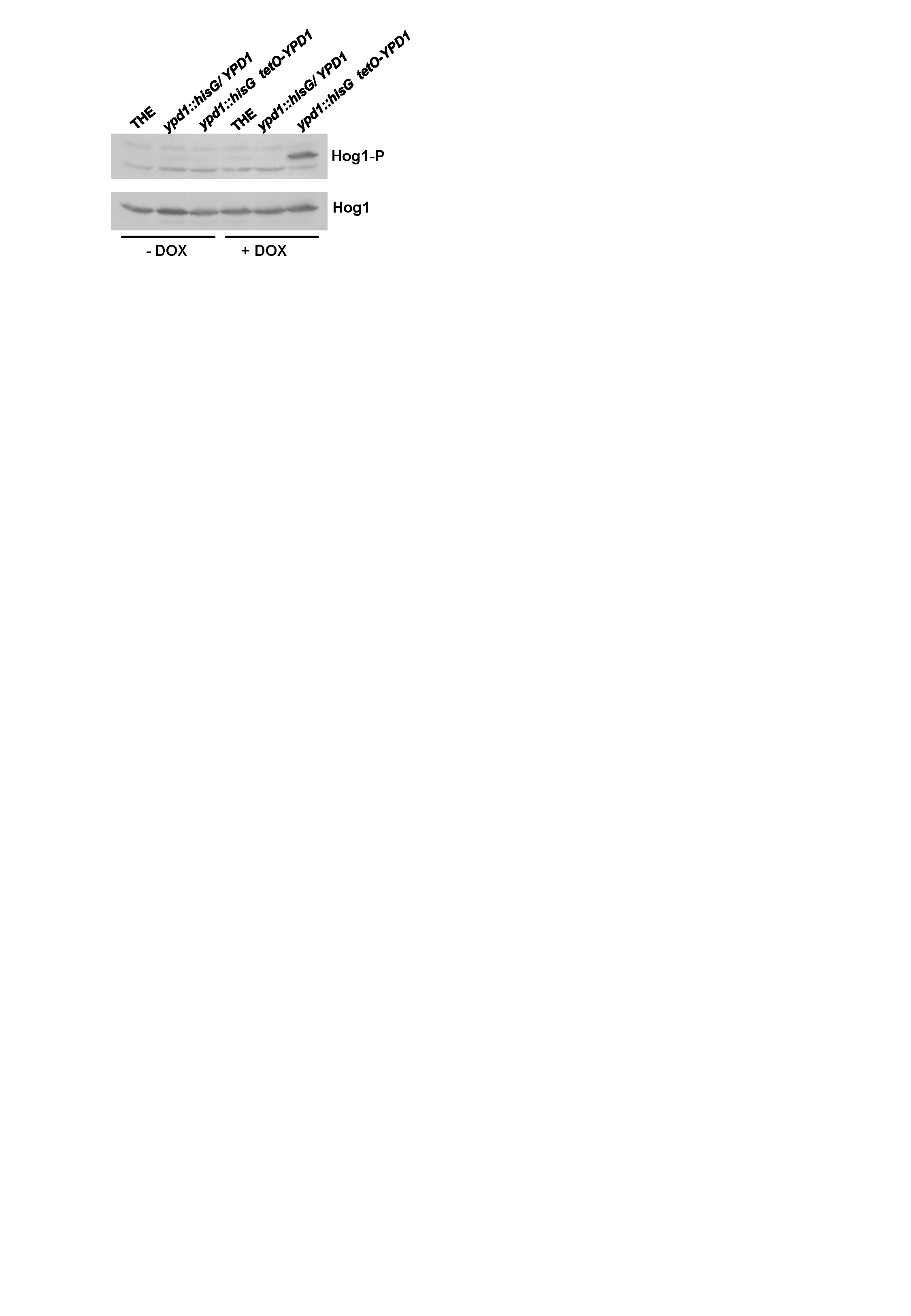

Supplement: S1 Fig — The indicated strains were treated or not with 20μg/ml doxycycline (DOX) for 1 h, and cell extracts were analysed for phosphorylated Hog1 by western blotting. Blots were probed for phosphorylated Hog1 (Hog1-P), stripped and reprobed for total Hog1 (Hog1). Hog1 phosphorylation is stimulated upon doxycycline-mediated reduction of YPD1 expression in tetO-YPD1 cells (JC1586), but not in the parental THE1 and THE1 ypd1::hisG (JC1420) strains following doxycycline treatment. (TIFF) [file ppat.1006131.s003.tiff]

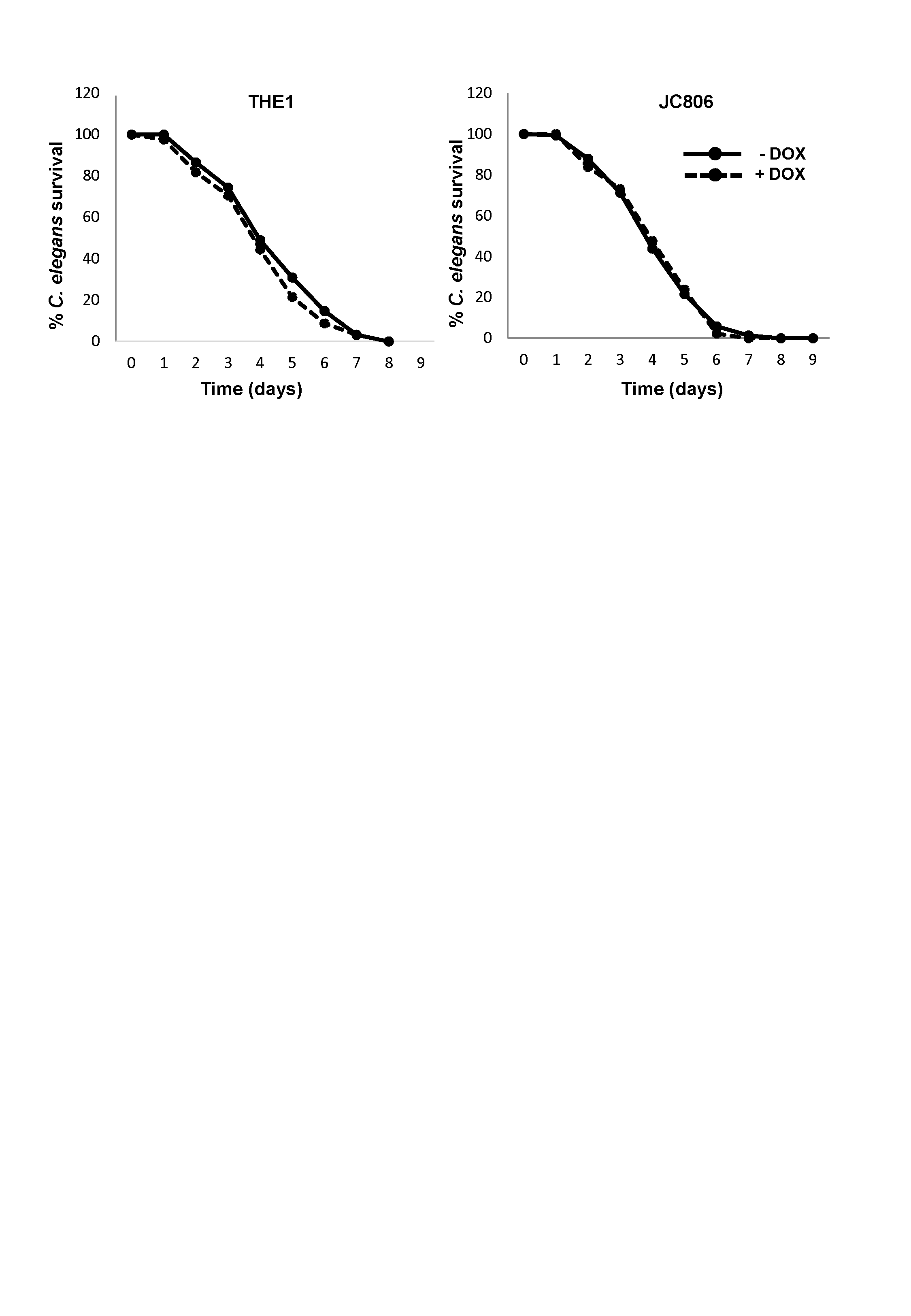

Supplement: S2 Fig — Nematodes were infected with wild-type THE1 or wild-type JC806 cells and transferred to liquid medium either with (+DOX) or without (-DOX) doxycycline. Doxycycline had no significant impact on nematode killing infected with either wild-type strain in (P>0.05). These data are from a single experiment representative of two independent biological replicates. (TIF) [file ppat.1006131.s004.tif]

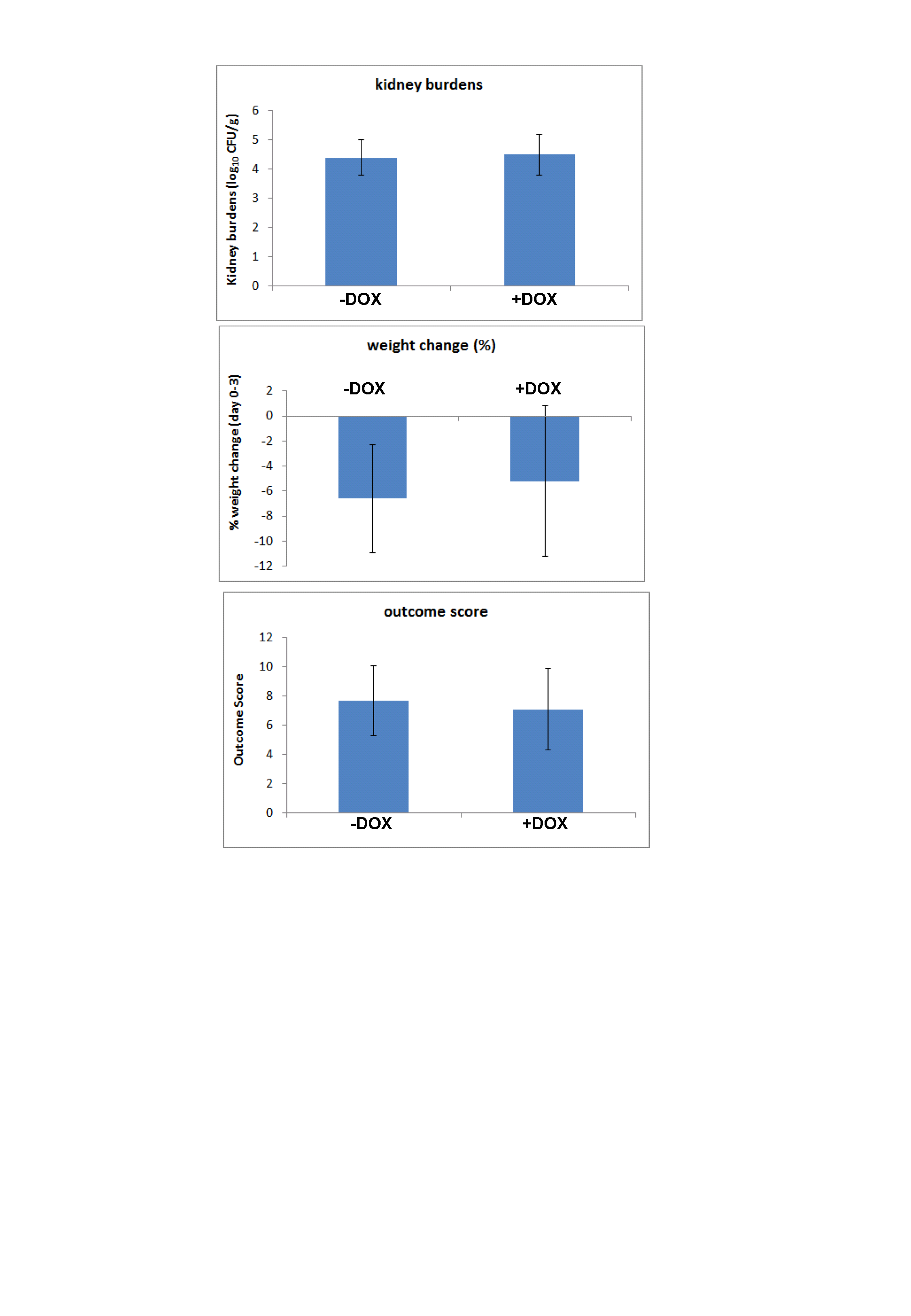

Supplement: S3 Fig — Kidney fungal burden measurements, percentage weight loss, and outcome score measurements of mice infected with wild-type C. albicans cells (SC5314) and administered doxycycline (+DOX) or not (-DOX). Comparison of +DOX and -DOX treated groups by Kruskal-Wallis statistical analysis found no significant differences for any of the three parameters. (TIF) [file ppat.1006131.s005.tif]

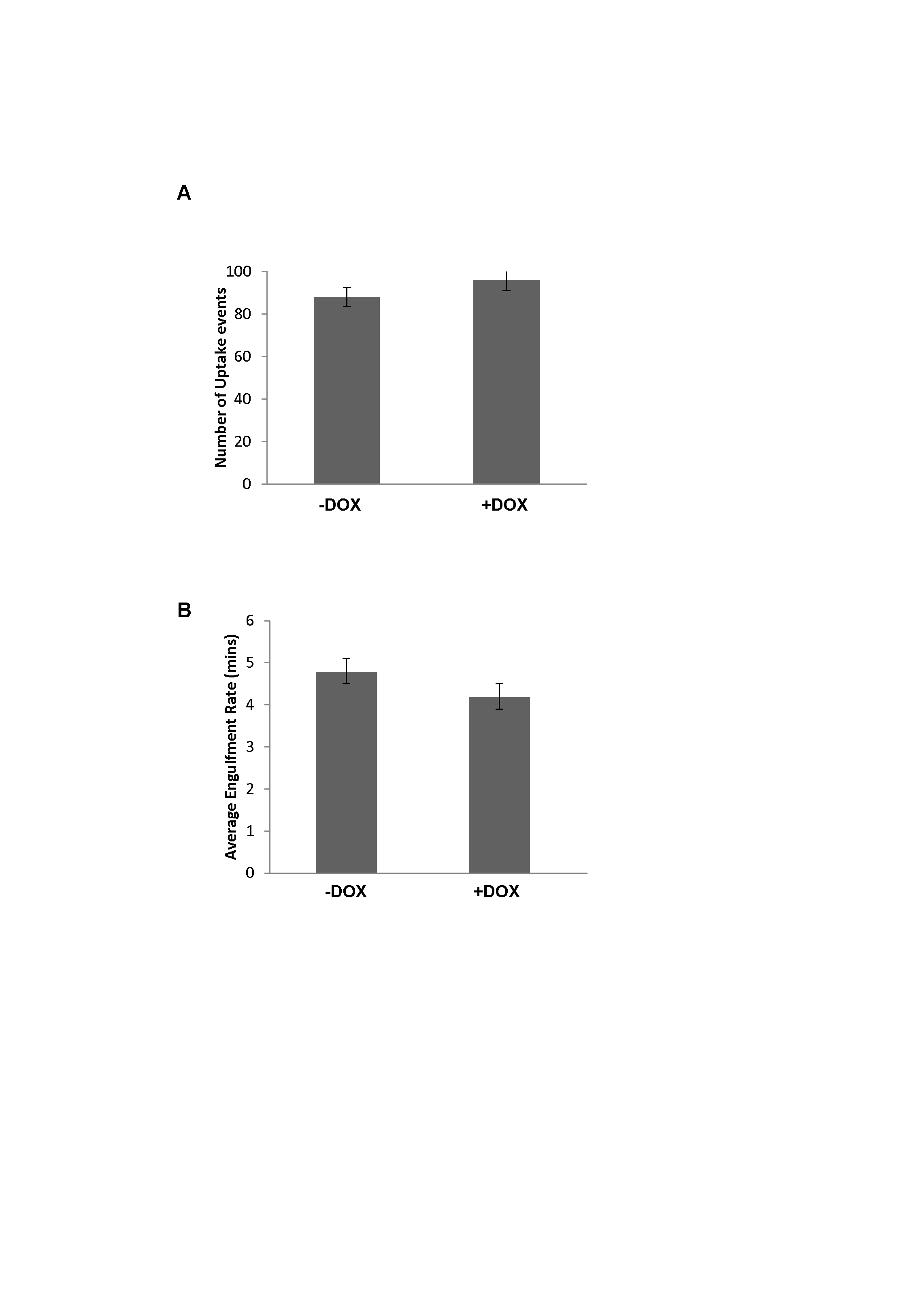

Supplement: S4 Fig — (A) Percentage uptake of tetOYPD1 cells grown in the presence (+DOX) or absence (-DOX) of doxycycline. No significant difference between uptake events + or − minus Dox by J774.1 macrophages after 6h co incubation was detected. (B) Engulfment time required for the ingestion of tetOYPD1 cells grown in the presence (+DOX) or absence (-DOX) of doxycycline. The bars represent the average time (minutes) taken for the complete engulfment of the cells by J774.1 macrophages. No significant differences between the rate of engulfment of fungal cells − or + Dox treatment were detected. (TIF) [file ppat.1006131.s006.tif]

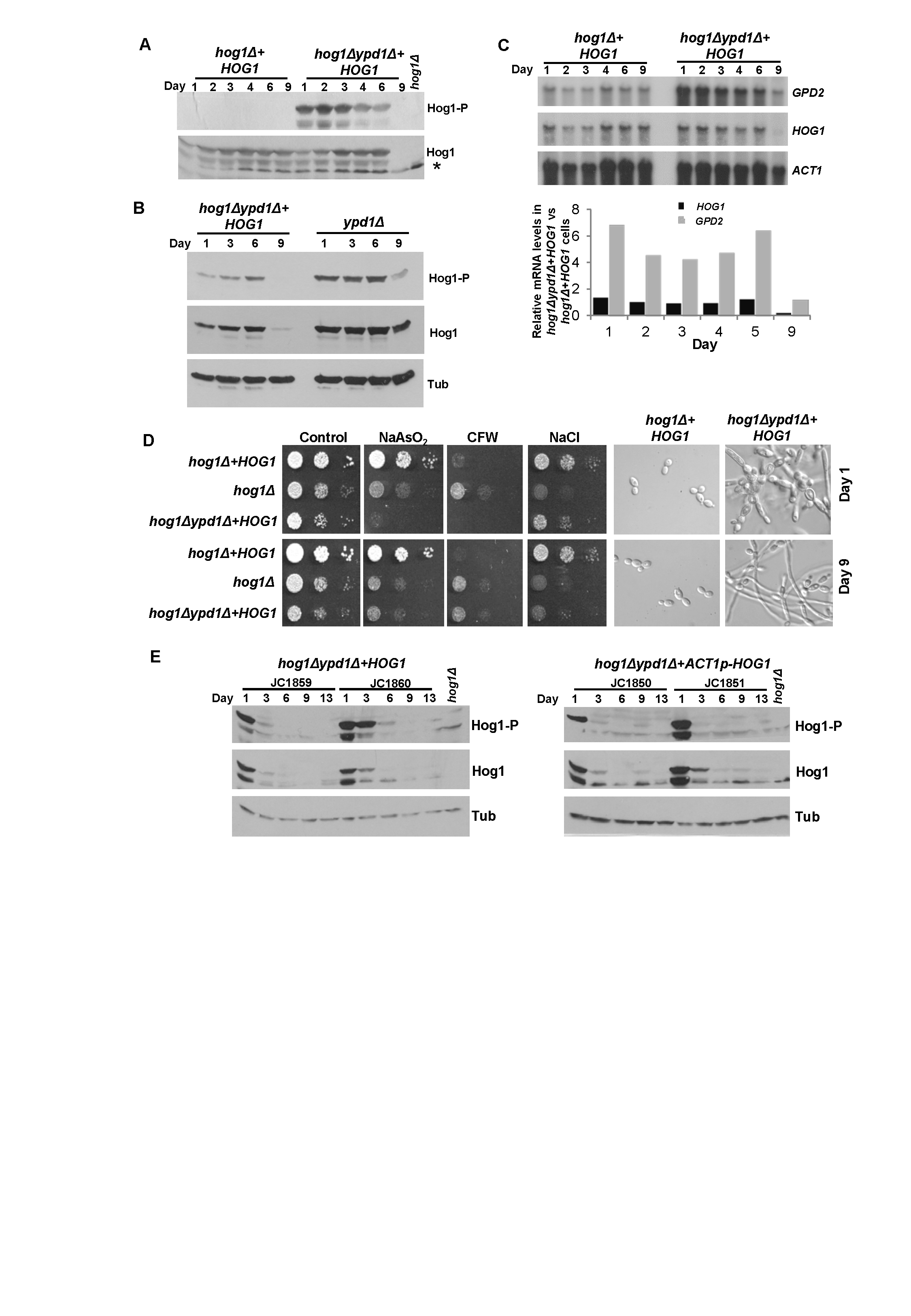

Supplement: S5 Fig — (A) Hog1 phosphorylation is not sustained in hog1Δypd1Δ+HOG1 cells over time and this is accompanied by a reduction in total Hog1 protein levels. Western blot analysis of whole cell extracts isolated from exponentially growing hog1Δ+HOG1 (JC52) and hog1Δypd1Δ+HOG1 (JC1478) cells taken from rich media plates after the number of days indicated. *indicates a non-specific band. (B) Comparison of Hog1 phosphorylation and Hog1 levels in hog1Δypd1Δ+HOG1 and ypd1Δ (JC2001) cells. Western blots were also probed for tubulin (Tub) in addition to phosphorylated (Hog1-P) and total Hog1 (Hog1). (C) GPD2 expression is not sustained in hog1Δypd1Δ+HOG1 cells and this correlates with a decline in HOG1 mRNA levels. Northern blot analysis of HOG1 and GPD2 expression in exponentially growing cells taken from rich media plates after the number of days indicated. The relative expression of HOG1 and GPD2 to the ACT1 loading control in is shown. (D) hog1Δ ypd1Δ+HOG1 cells gradually accumulate phenotypes characteristic of hog1Δ cells. Approximately 104 cells, and 10-fold dilutions thereof, of exponentially growing cells taken from rich media plates after Day 1 or Day 9 were spotted onto plates containing; NaAsO2 (1.5 mM), calcofluor white (30 μg/ml) and NaCl (0.5 M). Plates were incubated at 30°C for 24 hrs. Micrographs illustrating the morphology of hog1Δ+HOG1 and hog1Δypd1Δ+HOG1 cells at Day 1 and Day 9 are also shown. (E) Reduction of Hog1 levels at the RPS10 locus occurs independently of the HOG1 promoter sequence. Western blot analysis of whole cell extracts isolated from two freshly isolated independent hog1Δ ypd1Δ strains expressing HOG1 integrated at the RPS10 locus from its native promoter (JC1859, JC1860; left panel) or the ACT1 promoter ACT1p-HOG1 (JC1850, JC1851; right panel). Blots were processed as described in B. (TIF) [file ppat.1006131.s007.tif]
